# Genomic, Proteomic, and Biochemical Analyses of Oleaginous *Mucor circinelloides*: Evaluating Its Capability in Utilizing Cellulolytic Substrates for Lipid Production

**DOI:** 10.1371/journal.pone.0071068

**Published:** 2013-09-04

**Authors:** Hui Wei, Wei Wang, John M. Yarbrough, John O. Baker, Lieve Laurens, Stefanie Van Wychen, Xiaowen Chen, Larry E. Taylor, Qi Xu, Michael E. Himmel, Min Zhang

**Affiliations:** 1 Biosciences Center, National Renewable Energy Laboratory, Golden, Colorado, United States of America; 2 National Bioenergy Center, National Renewable Energy Laboratory, Golden, Colorado, United States of America; University of Missouri, United States of America

## Abstract

Lipid production by oleaginous microorganisms is a promising route to produce raw material for the production of biodiesel. However, most of these organisms must be grown on sugars and agro-industrial wastes because they cannot directly utilize lignocellulosic substrates. We report the first comprehensive investigation of *Mucor circinelloides*, one of a few oleaginous fungi for which genome sequences are available, for its potential to assimilate cellulose and produce lipids. Our genomic analysis revealed the existence of genes encoding 13 endoglucanases (7 of them secretory), 3 β-D-glucosidases (2 of them secretory) and 243 other glycoside hydrolase (GH) proteins, but not genes for exoglucanases such as cellobiohydrolases (CBH) that are required for breakdown of cellulose to cellobiose. Analysis of the major PAGE gel bands of secretome proteins confirmed expression of two secretory endoglucanases and one β-D-glucosidase, along with a set of accessory cell wall-degrading enzymes and 11 proteins of unknown function. We found that *M. circinelloides* can grow on CMC (carboxymethyl cellulose) and cellobiose, confirming the enzymatic activities of endoglucanases and β-D-glucosidases, respectively. The data suggested that *M. circinelloides* could be made usable as a consolidated bioprocessing (CBP) strain by introducing a CBH (e.g. CBHI) into the microorganism. This proposal was validated by our demonstration that *M. circinelloides* growing on Avicel supplemented with CBHI produced about 33% of the lipid that was generated in glucose medium. Furthermore, fatty acid methyl ester (FAME) analysis showed that when growing on pre-saccharified Avicel substrates, it produced a higher proportion of C14 fatty acids, which has an interesting implication in that shorter fatty acid chains have characteristics that are ideal for use in jet fuel. This substrate-specific shift in FAME profile warrants further investigation.

## Introduction

An essential challenge for next-generation cellulosic biofuels is overcoming the biomass recalcitrance and converting the biomass-derived sugars to biofuels [1], [2]. Microbial conversion of biomass to biofuels is an attractive route for biofuel development, because some microorganisms, including the model fungus *Mucor circinelloides*, are able to accumulate lipids by growing on the sugars released from the pretreatment of biomass; the lipids can be used as feedstock for biodiesel production [3], [4], catalysed by chemicals or lipases. Lipase from *M. circinelloides* has been commercialized, and its immobilization and utilization in fine chemistry and oil and fats modification have been reviewed recently [5], adding to the relevance of studying this species for developing biodiesel. The current state-of-the-art and prospects on microbial biodiesel production have been discussed by several recent reviews of the literature [6]–[10].

The suitability of *M. circinelloides* as a model lipogenic fungus is also based on the finding that it has a relatively broad associated molecular tool repertoire that includes genetic transformation using self-replicative plasmids [11] and mediation by *Agrobacterium* [12], electroporation [13], or polyethylene glycol [14]. In addition, its genome sequence is available, making possible genomic as well as secretomic approaches.

Furthermore, *M. circinelloides* is phylogenetically close to two other broadly studied lipogenic fungi, *Cunninghamella echinulata and Mortierella isabellina*, both of which are reported to accumulate high levels of lipids (60 to 70% w/w of dry cell mass) [15], [16]. Based on NCBI taxonomy databases (http://www.ncbi.nlm.nih.gov/sites/entrez?db=Taxonomy) and the literature [17], [18], these three species have a similar lineage (Eukaryota; Opisthokonta; Fungi; Fungi incertae sedis; Early diverging fungal lineages; Mucoromycotina; Mucorales). Insights gained from studies of *M. circinelloides* can benefit studies on *C. echinulata and Mort. isabellina*, inasmuch as the genomes of the latter two have not yet been sequenced.

Production of biofuels, including raw material for producing biodiesel, by growing oleaginous microorganisms on lignocellulosic substrates as carbon sources is promising because of the abundant renewable sources of raw materials [19], [20]. For this purpose, gaining insights to the cellulolytic capacity of a microorganism is often the first step for exploiting its innate potential to overcome the biomass recalcitrance [2]. So far, studies have demonstrated that *M. circinelloides* strains can utilize glucose, xylose [3], [4], [21], [22], acetic acid [23], and the hydrolysate of wheat straw after dilute-acid pretreatment [3]. However, no studies have been conducted to evaluate its capability for directly utilizing celluloses or the solid biomass residues after thermochemical pretreatment of cellulosic biomass. Promisingly, *M. circinelloides* strain NRRL 26519 was found to produce extracellular cellulases when grown on lactose and Sigmacell 50 and one 27 kDa endoglucanase was purified [24]. Meanwhile, other studies also purified two 43 and 47 kDa endoglucanases from another strain of this species [25], [26], but it is not yet known whether these secreted endoglucanases are common for different strains of this species, what other cellulases the species may have, and what potential this species has in directly utilizing cellulolytic substrates to produce lipids.

The major objective of this study is to evaluate *Mucor*'s cellulolytic capacity and assess its potential as a consolidated bioprocessing (CBP) microorganism capable of directly growing on cellulosic substrates and producing lipids. To achieve these goals, we have employed comprehensive, multi-directional approaches that include genomic, proteomic, and biochemical analyses to gain an understanding of the cellulose utilization machinery in *M. circinelloides*. We tested *M. circinelloides* ATCC 1216b (i.e., CBS 277.49), the specific strain for which the genome has been sequenced (http://genome.jgi-psf.org/Mucci2/Mucci2.home.html), for its ability to grow on plates incorporating cellulose as the sole carbon source. We searched the genome of *M. circinelloides* for genes putatively encoding cellulases and then used PAGE-fractionation with liquid chromatography/mass spectrometry (LC/MS) to catalog relevant, actually-expressed proteins in the secretome, with standard assays used to test for major cellulases in the secretome. The potential of *M. circinelloides* as a CBP microorganism for biomass-to-biofuel conversion was evaluated by its utilization of (1) pre-saccharified cellulosic substrates in the process of separate enzymatic hydrolysis and fermentation (SHF), and (2) un-pretreated microcrystalline cellulose (Avicel) with simple supplement of exoglucanase [i.e. cellobiohydrolase I (CBHI)] which was the major category of cellulase activity found to be undetectable in *M. circinelloides*.

## Materials and Methods

**Figure 1:**
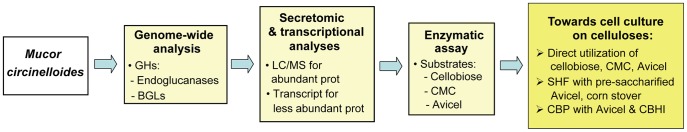
Schematic diagram of experimental approaches. The experiments were designed to progressively assess the capability of *M. circinelloides* in directly utilizing cellulosic substrates for its growth and lipid production. CBHI, cellobiohydrolase I; CBP, consolidated bioprocessing; CMC, carboxymethyl cellulose; GH, glycoside hydrolase; LC/MS, liquid chromatography/mass spectrometry; SHF, separate enzymatic hydrolysis and fermentation.

The overall experimental approaches are illustrated in Figure 1. The first step toward evaluating the cellulosic capability of *M. circinelloides* was to conduct a genome-wide search for the putative glycoside hydrolases (GHs).

### Cellulosic substrates

Carboxymethyl cellulose (CMC) and Avicel PH 101 were purchased from Sigma (catalogue nos. C4888 and 11365, respectively). The source of corn stover is described in the notes of Table 1.

**Table 1:** The different substrates used as carbon source in lipid production media.

Carbon sources	Description
(1) glucose	3% (w/v) in medium.
(2) Avicel cellulose	2.7% (w/v) in medium.
(3) DAPCS[1]	4.49% (w/v) pretreated corn stover (glucan content 60%) in medium, equivalent to 2.7% glucan (w/v) in medium.
(4) pre-saccharified Avicel[2]	Starting from 2×2.7% Avicel with a conversion rate of 85% to glucose. This pre-saccharification mixture was further mixed with equal volume of 2× lipid production medium, resulting in 2.6% (w/v) glucose in the final medium[2].
(5) pre-saccharified DAPCS[2]	Starting from 4.49% (w/v) pretreated corn stover with a conversion rate of 90% to glucose. This pre-saccharification mixture was further mixed with equal volume of 2× lipid production medium, resulting in 2.7% (w/v) glucose in the final medium[2].

**Notes:**

[1]. The corn stover (33A14) used in this study was harvested from the Kramer farm in Wray, Colorado. The milled corn stover powders were treated in the 4-L NREL Steam Gun reactor. The pretreatment conditions are: 160°C, 5 min, 2.0 wt% sulfuric acid. The composition of dilute acid-pretreated corn stover (DAPCS) was: Glucan 60.2%, xylan 4.8%, lignin 24.0%, galactan 0.7%, arabinan 2.1%.

[2]. Pre-saccharifications of Avicel or DAPCS were carried out as below: Starting from 2×4.49% (w/v) pretreated corn stover or 2×2.7% (w/v) Avicel by incubation for 3 days with a commercial “cellulase” (GC 220, Genecor/Danisco), loaded at 40 mg protein per g of glucan, at 50°C and pH 4.8 in 50 mM sodium citrate. The pre-saccharification mixture was boiled for 10 min, and then mixed with 2× lipid production (LP) medium to start the culturing.

### Genome-wide analyses of glycoside hydrolases and carbohydrate esterases

The putative glycoside hydrolases (GHs) and carbohydrate esterases (CEs) of *M. circinelloides* were annotated by applying the CAZymes (**c**arbohydrate-**a**ctive en**z**ymes) Analysis Toolkit (http://cricket.ornl.gov/cgi-bin/cat.cgi) [27] to the protein sequences deduced from the entire genome of *M. circinelloides* assembly v2.0 downloaded from US DOE Joint Genome Institute website (http://genome.jgi-psf.org/Mucci2/Mucci2.info.html). The approach used is based on a similarity search against the entire non-redundant protein sequences of the CAZy database (http://www.cazy.org/), supplemented with literature [24]–[26] retrieved from PubMed describing cellulase proteins identified in *M. circinelloides*. For the identified GHs of interest, BlastP was manually conducted to find the protein with the highest similarity, using the NCBI Blast analysis tool (http://blast.ncbi.nlm.gov/Blast.cgi).

### Microorganism and its maintenance and seeding culture

*M. circinelloides* strain ATCC 1216b (CBS 277.49) was used in this study and maintained in potato dextrose (PD) agar at 28°C. For all the liquid cultures, the seed culture prepared in PD broth was used as inoculum for the cultivation in each particular medium. Seed culture was produced by inoculating 100 mL of PD medium in a 300-mL Erlenmeyer flask with 100 µL of spore suspension that was collected from mature fungus grown on PD agar plates; alternatively, production of seed culture at larger scale was carried out by inoculating 300 mL of PD medium in a 1-L baffled flask with 300 µL of spore suspension that was collected from mature fungus grown on PD agar plates. In both cases, the seed culture was incubated at 28°C, 180 rpm for 2 d.

### Medium and culture for secretome protein collection

The growth medium for enzyme production, defined as lactose/yeast-extract medium, contains 10 g/L lactose, 1 g/L yeast extract, 2 g/L, KH_2_PO_4_, 1.4 g/L (NH_4_)_2_SO_4_, 0.6 g/L MgSO_4_·7H_2_O, 0.4 g/L CaCL_2_·2H_2_O, 0.005 g/L FeSO_4_·7H_2_O, 0.002 g/L MnSO_4_·H_2_O, 0.002 g/L ZnSO_4_, 0.004 g/L CoCL_2_·6H_2_O, and 0.015 g/L Tween 80. The pH of the medium was adjusted to 6.0 with 1M HCl.

A seed culture of 100 mL (as described above) was added to 1 L of enzyme production medium in a 2.8-L Erlenmeyer flask to initiate the cellulase production. After 5 d on a rotary shaker (180 rpm) at 28°C, the cells were removed from the culture broth by centrifugation (5000×g, 15 min), and the supernatant was used as the crude enzyme preparation for **(1)** assessment of secreted extracellular cellulase activities, and **(2)** production of the secretome protein preparation for direct and detailed analysis of individual secreted proteins, as described below.

### Secretome protein sample preparation

The samples of secreted protein used for direct biochemical analysis were prepared in parallel by two different methods.

#### Method 1: Concentrator extraction

The crude enzyme preparation (1 L) was first concentrated to 60 mL by ultrafiltration using an Amicon™ membrane with nominal MW cutoff of 10 kDa (EMD Millipore, Billerica, MA) and then further concentrated and exchanged into acetate buffer by five successive concentration and redilution cycles. Ten mL of the concentrated culture supernatant was placed in each of 6 Vivaspin™ centrifugal concentrators (Vivaproducts, Littleton, MA), and reduced, in each cycle, to half of the volume by centrifuging at 3500×g for 25 min. The ultrafiltrate was discarded and the retentate was returned to a total volume of 10 mL by addition of 5 mL of 50 mM acetate buffer, pH 4.8 buffer. After a total of five cycles of this process, the 6 final retentates were further centrifuged to 0.2 mL per concentrator. A total of 1.2 mL retentate was combined from the above 6 Vivaspin centrifugal concentrators, and the undissolved debris was pelleted by centrifugation at 1000 g for 10 min. The pellet was discarded and the supernatant was used as the “concentrator preparation” of secretome proteins. The total protein concentration of which was determined using the Pierce BCA Protein Assay kit (Thermo Scientific, Rockford, IL), followed by PAGE analysis to separate out individual proteins.

#### Method 2: Ethanol precipitation

The crude enzyme preparation (1 L) was concentrated to 150 mL by ultrafiltration with an Amicon™ membrane with nominal MWCO of 10 kDa (EMD Millipore). To precipitate the proteins, 480 mL of chilled ethanol was added drop-wise to the above concentrated solution to reach an ethanol concentration of 75% (v/v); the whole broth was then kept at −20°C overnight. The precipitate was pelleted by centrifugation at 26 000×g for 20 min and dissolved in 10 mL of 50 mM acetate buffer, pH 4.8. A single Vivaspin centrifugal concentrator (Vivaproducts, Littleton, MA) with MW cutoff of 10 kDa was used for desalting and concentrating the 10 mL of solution to half of the starting volume by centrifugation at 3500×g for 25 min. The filtrate was discarded and 5 mL of the same buffer was added to the retentate in the concentrator, which was then centrifuged again. After five cycles of this centrifugal process, the remaining 5 mL liquid was recovered from the concentrator, and the undissolved debris was pelleted by centrifuge at 1000×g. The pellet was discarded and the supernatant was collected and applied to a new centrifugal concentrator to be concentrated to 1 mL. This fraction was used as the “ethanol-precipitated” preparation of secretome proteins later subjected to protein concentration determination and PAGE analysis.

### SDS-PAGE analysis of proteins

The Pierce BCA Protein Assay (Thermo Scientific, Rockford, IL) was used to measure the concentration of protein in each of the protein extraction and analysis procedures. To run a SDS-PAGE (sodium dodecyl sulfate polyacrylamide electrophoresis) gel, the protein samples were denatured by being heated to 70°C for 10 min in the presence of a reducing agent (dithiothreitol) and SDS. Twenty µg of each of the denatured protein concentrates was loaded into each well of an Invitrogen NuPAGE Novex Bis-Tris Mini Gel. The gel was run at a constant 200 V for 35 min. After the electrophoresis, the gel was fixed with acetic acid/methanol solution, stained with Coomassie Blue overnight, and destained with deionized, distilled water for 7 h.

### Protein identification

Protein bands were excised from Coomasie Blue-stained SDS-PAGE gels and were identified using Nano LC-MS/MS peptide sequencing technology (ProtTech Inc., Norristown, PA). Two independent PAGE analyses were conducted to provide replicates for the protein extractions and subsequent LC/MS analysis. The obtained mass spectrometric data was used to search against the non-redundant protein database (NR database, NCBI) and the *M. circinelloides* dataset of protein sequence (http://genome.jgi-psf.org/Mucci2/Mucci2.info.html) with ProtTech's ProtQuest software suite. The output from the database search was manually validated.

### Growth of *Mucor* on cellulose-containing plates

The potential of *M. circinelloides* strain ATCC 1216b to degrade and utilize cellulose was assessed first by growth on CMC or Avicel agar plates. The mineral growth medium in the plates consisted of: 10 g/L CMC or Avicel cellulose, 2 g/L of K_2_HPO_4_, 1.4 g/L (NH_4_)_2_SO_4_, 0.6 g/L MgSO_4_·7H_2_O, 0.4 g/L CaCL_2_·2H_2_O, 0.005 g/L FeSO_4_·7H_2_O, 0.002 g/L MnSO_4_·H_2_O, 0.002 g/L ZnSO_4_, 0.004 g/L CoCL_2_·6H_2_O, and 15 g/L agar. The plates were incubated at 28°C for 5 d.

### Extracellular enzyme activity assays

To test the extracellular cellulase activities produced by *M. circinelloides*, the crude enzyme solution prepared as described above was incubated with 1.0 wt % of either CMC or Avicel at 50°C and pH 4.8 (0.1 M acetate buffer) for 1 h. The release of sugars from CMC or Avicel was determined by HPLC using a BioRad Aminex HPX-87P column (85°C, water at 0.6 mL/min as mobile phase) with refractive-index detection.

### Effect of growth carbon source on lipid production: glucose *vs.* cellobiose

A 90-mL seed culture of *M. circinelloides* (prepared as described above) was equally divided into 3 aliquots (30 mL for each aliquot). The cells in each aliquot were pelleted by centrifugation and the supernatant discarded; the cell pellet from one aliquot (containing very little of the seeding medium) was then used to inoculate another 300 mL of media containing different carbon sources (as described below).

All of the lipid-production media contained (g/L): (NH_4_)_2_SO_4_, 0.5; KH_2_PO_4_, 7.0; Na_2_HPO_4_, 2.0; MgSO_4_· 7H_2_O, 1.5; CaCl_2_·2H_2_O, 0.1; FeCl_3_·6H_2_O, 0.08; ZnSO_4_·7H_2_O, 0.001; CuSO_4_·5H_2_O, 0.0001; CoCl_2_·H_2_O, 0.0001; MnSO_4_·5H_2_O, 0.0001; and yeast extract, 0.5, supplemented with the appropriate amount of carbon source. The carbon sources used were: **(1)** no sugar addition (control), **(2)** glucose (3%, w/vol.), and **(3)** cellobiose (2.85%, w/vol.). All cultures were grown in 1-L Erlenmeyer flasks that contained 300 mL of the above medium. The flasks were incubated in a rotary shaker at 180 rpm and 28°C for 7 d. Duplicate fermentations were run for each of the carbon sources.

The initial pH value of the medium in each flask used for the carbon-source survey was pH 6.0. Aliquots of initial time point samples (2 ml for each) were analyzed by HPLC, in order to track the types and quantities of sugar residues that were brought over with the inoculation pellets taken from the seeding culture. The ending time-point samples were centrifuged at 5000×g for 10 min, and the resulting supernatants were used to measure the pH, while the cell pellets were used for .the determinations of fungal mass and lipid contents.

### Evaluation of *Mucor* in separate hydrolysis and fermentation using pre-saccharified substrates

A 300-mL seed culture (prepared as described above) was equally distributed into 10 aliquots (30 mL for each aliquot). The cells of each aliquot were pelleted by centrifugation and the supernatant discarded; the cell pellet from one aliquot (containing very little of the seeding medium) was then used to inoculate 300 mL of lipid-production medium containing one of five different carbon sources (as described below).

The lipid-production medium, as described above in the section on “Effect of growth carbon source on lipid production: glucose *vs.* cellobiose”, was supplemented with the appropriate amount of each different carbon source. The carbon sources used were: **(1)** glucose, **(2)** model cellulosic material, i.e., Avicel (PH 101, Sigma-Aldrich), **(3)** dilute acid-pretreated corn stover (DAPCS), **(4)** pre-saccharified Avicel, and **(5)** pre-saccharified DAPCS. The pre-saccharification of Avicel and DAPCS, and amounts of each substrate used are described in Table 1. All cultures were grown in 1-L Erlenmeyer flasks that contained 300 mL of one of the above media. The flasks were incubated in a rotary shaker at 180 rpm and 28°C. Duplicate fermentations were run for each of the carbon sources.

The initial pH value of the medium in each flask used for the carbon-source survey was pH 6.0. Culture aliquots of 15 mL were taken daily to measure the pH and optical density, as well as for bioimaging analyses. The ending time point samples were centrifuged at 5000 g for 10 min, and the resulting cell pellets were used for .the determinations of fungal mass and lipid content.

### Purification of cellobiohydrolase I

The commercial CBHI of *Trichoderma longibrachiatum* was obtained from Megazyme International (Wicklow, Ireland; catalogue No. E-CBHI). The purity of the commercial CBHI was checked by SDS-PAGE, and the purchased preparation was further purified as described below. About 60 mg of CBHI from Megazyme (Wicklow, Ireland) was dissolved in 1.5 M (NH_4_)_2_SO_4_ - 20 mM Bis-Tris, pH 6.5, loaded onto a Resource Iso 1-mL hydrophobic interaction chromatography (HIC) column (GE Healthcare), and eluted with a 10-column-volume linear gradient from 1.5 M to 0 M (NH_4_)_2_SO_4_ in the same buffer. The eluted CBHI was further run over a Superdex 75 26/60 size exclusion column (GE Healthcare, Piscataway NJ) using 20 mM sodium acetate, pH 5.0, 100 mM NaCl. The major UV 280 peak was pooled. Fractions were assayed for activity against 2 mM 4-nitrophenyl-β -D-lactopyranoside (p-NPL) dissolved in 50 mM sodium acetate, pH 5.0. Twenty-five microliters of each fraction was added to 125 µL substrate solution in a 96-well plate, incubated at 45°C for 30 min, and quenched by addition of 25 µL of 1M NaCO_3_. Absorbance at 405 nm was then read on a Spectramax 96-well photospectrometer. Measured absorbances were compared to a standard curve prepared by reading absorbance of 4-nitrophenol at concentrations ranging from 0 to 250 µM in the same buffer. Fractions exhibiting p-NPL activity were evaluated by SDS-PAGE. CBHI fractions were pooled and concentration determined by the BCA protein assay (Pierce, Rockford, IL) according to manufacturer's instructions.

### Consolidated bioprocessing of cellulosic substrates by *Mucor* supplemented with cellobiohydrolase I

The preparation of 100 mL of seed culture in PD broth was as described above. Eight aliquots (3 mL for each aliquot) were taken from the seed culture. The cells of each aliquot were pelleted by centrifugation and the supernatant discarded; the cell pellet from one aliquot (containing very little of the seeding medium) was then used to inoculate 33 mL of media containing the different carbon sources described below.

The composition of basal lipid-production medium used for the CBP was the same as that for the SHF. Each culture was grown in 250-mL Erlenmeyer flasks that contained 33 mL of one of the following three types of media. The carbon sources and enzymes used in 33 mL culture were: (**1)** basal medium alone, serving as no-carbohydrate control; **(2)** basal medium plus 1.0 g glucose; **(3)** basal medium plus 0.9 g Avicel and 20 mg purified commercial CBHI of *T. longibrachiatum*. The flasks were incubated in a rotary shaker at 180 rpm, 28°C for 10 d. Duplicates were run for each of the carbon sources.

The initial pH value of the medium in each flask used for the carbon-source survey was pH 6.0. After 12 d cultivation, an aliquot (900 µL) of the culture broth was taken out for Nile red staining for cellular distribution of lipids. The rest of culture broth was centrifuged at 5000 g for 10 min, and the supernatant was used for pH and HPLC analyses, and the resulting pellets were used for the determinations of lipid contents in each culture.

### Nile red staining for cellular distribution of lipids

Intracellular imaging of the lipid distribution was performed using a Nikon Eclipse E800 laser scanning confocal microscope with a spectrometer. The culture broths (1 mL for each sample) were centrifuged, and the pellets were washed twice, and then resuspended in 900 µL DI water. Subsequently, these broths were mixed with 100 µL stock solution of 100 µg/mL concentration of Nile Red (9-diethylamino-5-benzo[α]phenoxazinone) obtained from Sigma and dissolved in dimethyl sulfoxide (DMSO), resulting in a final Nile Red concentration of 10 µg/mL. These mixtures were allowed to stain for 10 min in the dark, and then were centrifuged, and the pellets were washed with DI water, followed by resuspension in 1 mL DI water. The stained yeast and fungal cells were imaged with the laser scanning confocal microscope with an excitation wavelength at 488 nm and a notch filter rejecting 488 nm and 514 nm for the spectral imaging. For this imaging work, the gain on the detecting PMTs was kept constant, as was the pixel dwell time.

### Measurement of Avicel residue in Avicel-fungal cell pellet

The Avicel residue from the *Mucor* culture growing on Avicel was mixed with fungal cells after centrifugation. The collected Avicel-fungal cell pellet was freeze-dried and weighed. The amount of Avicel contained in the pellet was determined by enzymatic digestions using GC220 cellulase enzyme (Genencor, Rochester, New York). The enzymatic digestions were performed in 50 mL of 50 mM citrate buffer, pH 4.8, containing 1% (w/v) substrate (i.e. Avicel-fungal cell mix) and 60 mg GC220 protein per g substrate. The enzymatic reaction mixture contained Na-azide (0.04% w/v) to prevent microbial growth. The reaction mixture was put into 125-mL Erlenmeyer shake flasks and incubated at 50°C and 130 rpm for 96 h according to NREL LAP (Laboratory Analytical Procedure) 009 [28]. No additional β-D-glucosidase (BGL) was added. The total glucose released was measured by HPLC, and was used to calculate the corresponding amount of Avicel contained in the Avicel-fungal cell mix. In parallel, freeze-dried Avicel was put through the same procedure as a standard for comparison.

### Lipid determination

The cell biomass grown on lipid production medium with glucose as carbon source was harvested by centrifugation, freeze dried, and then used for determination of lipid content using a one-step extraction and esterification procedure [29]. A mixture of 0.2 mL chloroform-methanol (2∶1 v/v) and 0.3 mL HCL-methanol (5% v/v) was added to 7 to 11 mg of lyophilized biomass. The mixture was then vortexed in a sealed glass vial and heated to 85°C to simultaneously solubilize and transesterify the lipids. The resulting fatty-acid methyl esters (FAMEs) were extracted with 1 mL hexane at room temperature for 1 h and analyzed by gas chromatography with flame-ionization detection (GC-FID) on an Agilent 6890N chromatograph using a HP5-MS column (Agilent, USA). The fungal mass samples were analyzed in the presence of 250 ug of C^13^-FAME as a quantitative internal standard. Quantification of the FAMEs was based on integration of individual fatty acid peaks in the chromatograms, peak areas being compared with those for a 5-point calibration curve of a mixed even-carbon-chain FAME standard mixture of 14 individual fatty acids (C8–C24, Sigma; catalog no. 18918). The total lipid content was calculated as the sum of the even numbered FAMEs.

## Results and Discussion

### Genome-wide search for cellulases in *M. circinelloides*

We annotated the CAZy proteins in *M. circinelloides* using the CAZymes Analysis Toolkit [27]. As summarized in Table 2, out of the 11719 proteins annotated in the genome of M. *circinelloides*, 259 and 126 were predicted to be GHs and CEs, respectively. The detailed list of these GHs and CEs, along with other CAZymes, is presented in Table S1.

**Table 2:** Overall genome feature of *M. circinelloides*.

Genome size	36.6 Mb
Haploid chromosomes	10
Total no. of proteins	11719
Total no. of GHs	259
Total no. of CEs	126

The full annotation of glycoside hydrolases (GHs) and carbohydrate esterases (CEs) is listed in Table S1.

GH families 5, 6, 7, 8, 9, 12, and 45 are known to include endoglucanases [30]. In the *M. circinelloides* genome, we found four GH5, two GH8, four GH9, and three GH45 enzymes; whereas GH families 6, 7, and 12 are unrepresented (Table 3).

**Table 3:** List of predicted endoglucanases and β-D-glucosidases (BGLs) in *M. circinelloides* genome.

Protein ID	Protein names	SP[1]	Theoretical MW	BlastP match	
				Species[2]	Protein
**Endoglucanase (GH5)**					
181082	cellulase	1-23	55.2 kDa	*A. oryzae*	XP_001727092
128967	endo-β-1,4-glucanase C	no SP	47.2 kDa	*P. chry. Wis.*	XP_002557627
14085	endo-β-1,4-glucanase C	not full length	32.8 kDa	*L. bicolor*	XP_001886234
39521	cellulase	no SP	80.8 kDa	*S. lacry. var. lacry.*	EGO01133
**Endoglucanase (GH8)**					
155404	Endoglucanase Y	no SP	24.0 kDa	*R. cham.*	CBL17008
108819	Endoglucanase Y	no SP	41.1 kDa	*R. cham.*	CBL17009
**Endoglucanase (GH9)**					
156165	Cel9A	1-19/1-23	59.8 kDa	*Ph. chryso.*	AAM22492
153977	Cel9A	no SP	60.0 kDa	*Ph. chryso.*	AAM22493
114542	Cel9A	1-20	72.8 kDa	*Ph. chryso.*	AAM22494
104610	β-1,4-endoglucanase	1-22	75.8 kDa	*E. andrei*	ACE75511
**Endoglucanase (GH45)**					
BAD95808[3]	β-1,4-endoglucanase	1-22	34.3 kDa	*Rhizopus oryzae*	BAC53988
BAD95809[3]	β-1,4-endoglucanase	1-22	39.3 kDa	*Rhizopus oryzae*	BAC53988
157172	Barwin-like endoglucanases	1-23	18.4 kDa	*F. mediter*	EJD02345
**β-D-glucosidase (GH3)** [4]					
33615	BGL	1-25	78.4 kDa	*Rh. miehei*	CAP58431
38405	BGL	1-25	78.6 kDa	*Rh. miehei*	CAP58431
153684	BGL	no SP	75.1 kDa	*Rh. miehei*	CAP58431

The listed enzymes include those of secretory type with signal peptides (SP) predicted and non-secretory (intracellular) types with no SP predicted.

**Notes:**

[1]. Signal peptides (SP) were predicted by TargetP and PSORT, which usually predicted the same SP. If different SPs were predicted, both SPs were presented (first TargetP and then PSORT's prediction).

[2]. Full names of species: *A. oryzae*, *Aspergillus oryzae*; *F. mediter.*, *Fomitiporia mediterranea*; *P. chry. Wis., Penicillium chrysogenum* Wisconsin 54-1255; *L. bicolor, Laccaria bicolor* S238N-H82; *S. lacry. var. lacry., Serpula lacrymans* var. lacrymans; *R. cham., Ruminococcus champanellensis; Ph. chryso., Phanerochaete chrysosporium; E. andrei, Eisenia andrei; Rh. miehei, Rhizomucor miehei*.

[3]. These two GH45 endoglucanases were previously purified from *M. circinelloides* by Baba et al. (2005). Their genomic sequence is located in the genome database of Mucor_circinelloides_v2_masked_scaffolds, scaffold_02, position 1045611 to 1046983.

[4]. These three BGLs are similar: Protein with ID 33615 has 73% and 60% similarity in amino acid sequence with 38405 and 153684, respectively.

Out of these possible endoglucanases, one GH5 (protein ID 181082), three GH9 (protein ID 156165, 114542, and 104610), and three GH45 (protein IDs BAD95808, BAD95809, 157172) are of special interest as they are predicted to contain a signal peptide (SP) suggesting that they are secretory and may contribute to the extracellular hydrolytic activity observed on CMC.

In the CAZy system, BGLs (EC 3.2.1.21) are distributed in GH families 1, 3, 9 and 116 (www.cazy.org). In our genome-wide analysis, three BGLs (GH3) were identified, of which two were predicted to be extracellular (protein IDs 33615 and 38405). This is consistent with previous reports of BGL from *M. circinelloides*, although it was not specifically identified and characterized in those studies [24], [31].

### Identification of relatively abundant extracellular proteins from major PAGE bands

**Figure 2:**
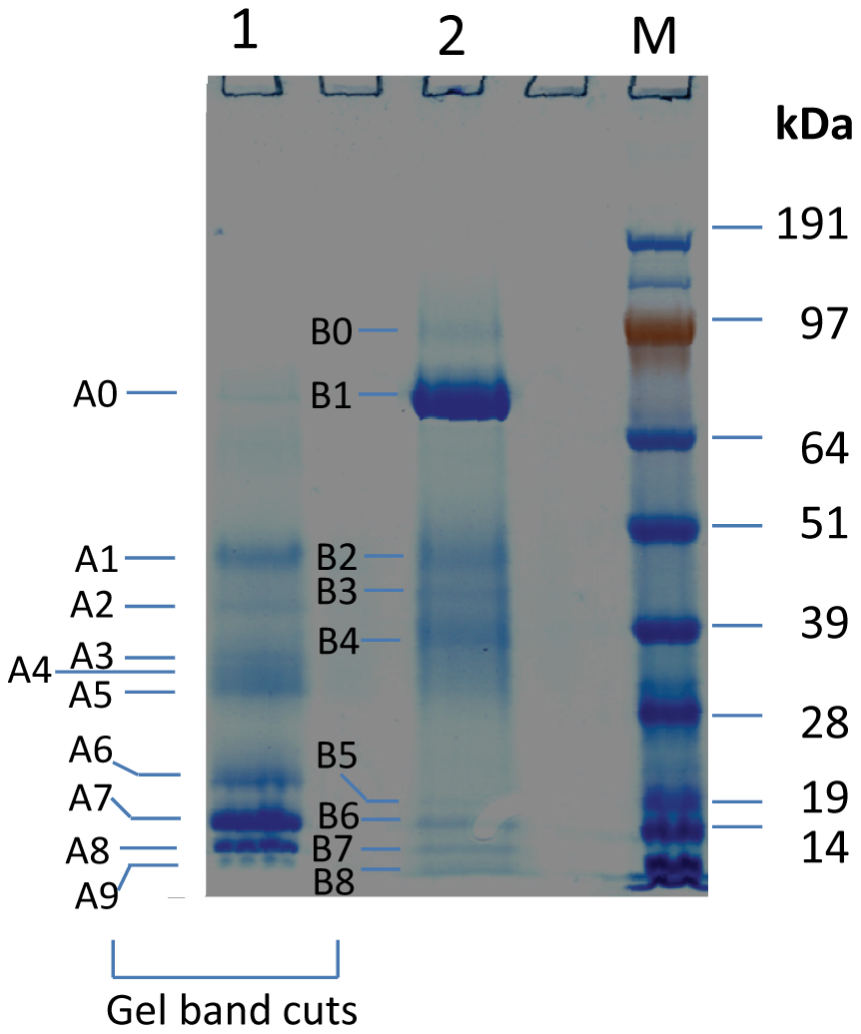
PAGE analysis of the secretome proteins of *M. circinelloides*. Lane 1. Total extracellular proteins by direct ultrafiltration and concentration (described as concentrator-prepared), with the gel bands A0–A9 being cut for LC/MS peptide identification. Lane 2. Total extracellular proteins by direct ultrafiltration and ethanol precipitation (described as ethanol-prepared), with the gel bands B0–B7 being cut. M, molecular mass markers in kilodaltons (kDa). All proteins were loaded equally at 20 µg per lane.

The secretome protein fractions were prepared from culture filtrates of 5-d growth on lactose/yeast-extract media, using the concentration and the ethanol precipitation procedures discussed above. The secretome proteins extracted by each procedure were fractionated by SDS-PAGE (Figure 2). The major bands (A0–A9 in the concentrator-prepared secretome samples and B0–B8 in the ethanol precipitation preparations) were manually excised from the gel and digested with trypsin followed by peptide identification using LC/MS. The detailed list of the peptide sequences and proteins identified in the bands of A0–A9 and B0–B8 is presented in Table S2.

The identified relatively abundant secretome proteins are listed in Table 4. First, 19 proteins were identified in the secretome protein preparation of concentrator method (see bands A0–A9 in Figure 2), whereas 14 proteins were identified in the secretome protein preparation of ethanol precipitation method (see bands B0–B8). In combination, these two extraction methods identified a total of 25 relatively abundant proteins, all of which were further confirmed to be secreted proteins by the prediction of fused signal peptides.

**Table 4:** List of proteins identified in the major SDS-PAGE gel bands of secretome proteins of *M. circinelloides*.

Protein ID	Protein names[1]	CAZy family	SP[2]	Theoretical MW	Gel bands located[3]	
**CAZy family proteins (1 CE and 7 GHs)**						
155048	polysaccharide deacetylase	CE4	1-21	46.7 kDa		B3
86506	osmotin, thaumatin-like protein	GH2	1-20	23.6 kDa	A6	
38405	BGL	GH3	1-25	78.6 kDa		B0
156167	glucan 1,4-alpha-glucosidase	GH15/CBM21	1-32/1-19	64.9 kDa	A0	B1
156806	concanavalin A-like lectin/glucanase	GH16	1-24/1-18	34.1 kDa	A3, A4	
181379	polygalacturonase	GH28	1-22/1-32	41.4 kDa		B3
BAD95808	β-1,4-endoglucanase	GH45	1-22	34.3 kDa	A2	
157172	Barwin-like endoglucanases	GH45	1-23	18.4 kDa	A7, A8	
**Other proteins (6)**						
156269	pepsin A		1-21	42.3 kDa	A1, **A2**	
155817	lipase		1-27	41.5 kDa	A5	
155115	phosphoesterase		1-19/1-21	37.6 kDa	**A1, A2**	**B2, B3**
84529	lipase		1-19/1-18	31.9 kDa		B2-3, **B4**
115625	endoribonuclease		1-16	28.5 kDa		**B4**
155263	MD-2-related lipid-recognition		1-18	21.3 kDa		B7
**Function unknown proteins (11)**						
157488	unassigned function		1-21/1-17	30.7 kDa	A7	B5
156010	unassigned function		1-22	30.3 kDa	A8	B7
115037	unassigned function		1-20	20.7 kDa	**A4**, **A5**, A7	B3, **B4**, B5-6
92193	unassigned function		1-19	19.5 kDa	A6, A7	
156218	unassigned function		1-19	18.9 kDa	A8	
155646	unassigned function		1-22	18.9 kDa	A3-5, **A6**, **A7**, A8	B6
157923	unassigned function		1-18	18.9 kDa	A7, A8	
184762	unassigned function		1-18/1-15	17.4 kDa	A7, A9	
157285	unassigned function		1-22	16.1 kDa	A8	
156103	unassigned function		1-22	14.9 kDa	A6-7, **A8**, A9	B6, **B7**, B8
191377	unassigned function		1-18	11.7 kDa	A6, **A7**, A8	B5, B6

**Notes:**

[1]. The **c**arbohydrate-**a**ctive en**z**yme (CAZy) family proteins are listed in the alphabetical order of CAZy families. Other proteins are listed in the order of molecular weight (MW) of proteins.

[2]. Signal peptides (SPs) were predicted by TargetP and PSORT, as described in the notes in Table 3.

[3]. The gel band locations were described in the legend of Figure 2. In short, bands A0–A9 are from concentrator-prepared proteins (from which a total of 18 proteins were identified), while bands B0–B8 are from ethanol-prepared (from which a total of 13 proteins identified). The bold bands represent the major band(s) from which the proteins were detected, with relatively higher numbers of peptides identified by LC/MS.

It is noteworthy that six secretory proteins (ID 155048, polysaccharide deacetylase; ID 38405, BGL; ID 181379, polygalacturonase; ID 84529, lipase; ID 115625, endoribonuclease; and ID 155263, MD-2-related lipid-recognition protein) were identified ONLY in the secretome proteins precipitated by ethanol, suggesting that their initial concentrations are low in the secretome and that they were selectively concentrated by the ethanol precipitation step. The strongest band in the ethanol-precipitated secretome proteins is that of the glucan 1,4-α-glucosidase (i.e. α-amylase, an enzyme involved in utilization of starch) that contains GH15/CBM21 domains (protein ID 156167; Figure 2, and Table 4, B1 band; CBM, cellulose binding module), suggesting that ethanol precipitation is an efficient method for extracting this enzyme.

A comparison between Tables 3 and 4 reveals that out of the 7 genome-predicted extracellular endoglucanases, two were identified in the relatively strong gel bands of the secretome protein extraction; these were GH45 β-1,4-endoglucanase (ID BAD95808) and GH45 Barwin-like endoglucanase (ID 157172). Meanwhile, out of the two genome-predicted extracellular BGLs, one was identified in the secretome (ID 38405), and has a high similarity with the extracellular BGL1 of *Trichoderma reesei*.

In the cases of the 5 other extracellular endoglucanases and 1 extracellular BGL that were predicted by our genome-wide GH search, but not detected in either extract from the secretome, we detected their transcripts in extracted mRNA preparations (data not shown). The presence of their mRNAs suggests that these proteins, while expressed, may have gone undetected in the secretome because of low abundance. Either their proteins in the secretome are located in “gap areas” between the excised strong bands, or their protein abundance is below the detection limit if they are located in the excised strong bands. This issue may provide an opportunity for increasing the cellulolytic capacity of the strain, by increasing the level of expression of these proteins.

### Secretome proteins with accessary cellulolytic functions or no clearly defined functions

In addition to the major cellulases discussed above, the polysaccharide deacetylase identified in the secretome (Protein ID 155048; CE4, Table 4) is also a valuable accessory enzyme for enhancing the overall cellulolytic capacity. The presence of this protein in the secretome suggests that *M. circinelloides* can likely cleave acetyl groups from the xylan backbone of the “hemicellulose” fraction in biomass. In view of the report that *M. circinelloides* can utilize acetate and in so doing achieve a higher lipid yield when acetate is combined with other carbon substrates [32], polysaccharide deacetylase is another valuable target protein for enhanced expression to increase usefulness of *Mucor* for biomass conversion.

In addition, there are 11 proteins for which functions are unknown, accounting for nearly half of the identified proteins in the secretome. Their functional study in future may lead to identifying novel proteins that also may facilitate cellulolysis.

### Growth of *M. circinelloides* on cellulose agar plates

**Figure 3:**
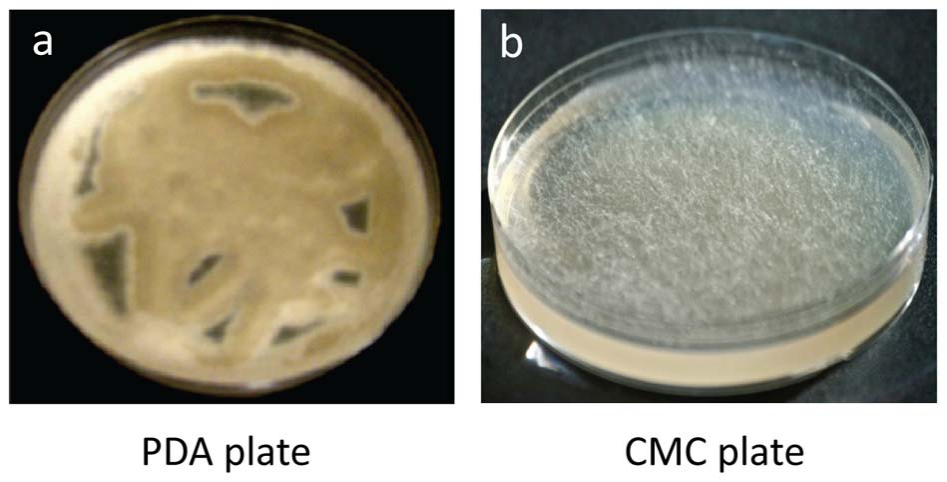
Growth of *M. circinelloides* on plates. They were grown on potato dextrose (PD) and carboxymethyl cellulose (CMC) agar plates for 5 d.

Growth on cellulose agar plates was used to examine the cellulolytic activity of the microbial strains. As Figure 3b shows, *M. circinelloides* grew on CMC cellulose plates; however, it grew only marginally on an Avicel plate (image not shown); our test showed that *M. circinelloides* also had marginal growth on water agar, which likely contributes to the strain's marginal growth on Avicel plate. These observations suggest that the *M. circinelloides* strain studied has some glycoside hydrolase activity, but probably does not express a “complete” cellulase system.

Such observation is consistent with our above described genome-wide search results that revealed the existence of 7 putative endoglucanses in the genome of *M. circinelloides*. More specifically, our above-described secretomic analysis identified endoglucanase (BAD95808) in the major SDS-PAGE bands, and this protein was reported to have enzymatic activity against both CMC (i.e. CMCase) and Avicel (i.e. Avicelase) [25], which likely contributes to the observed *M. circinelloides* growth on CMC cellulose plates and marginal growth on Avicel plates we have noted above.

### Extracellular cellulase activity of *M. circinelloides*

**Figure 4:**
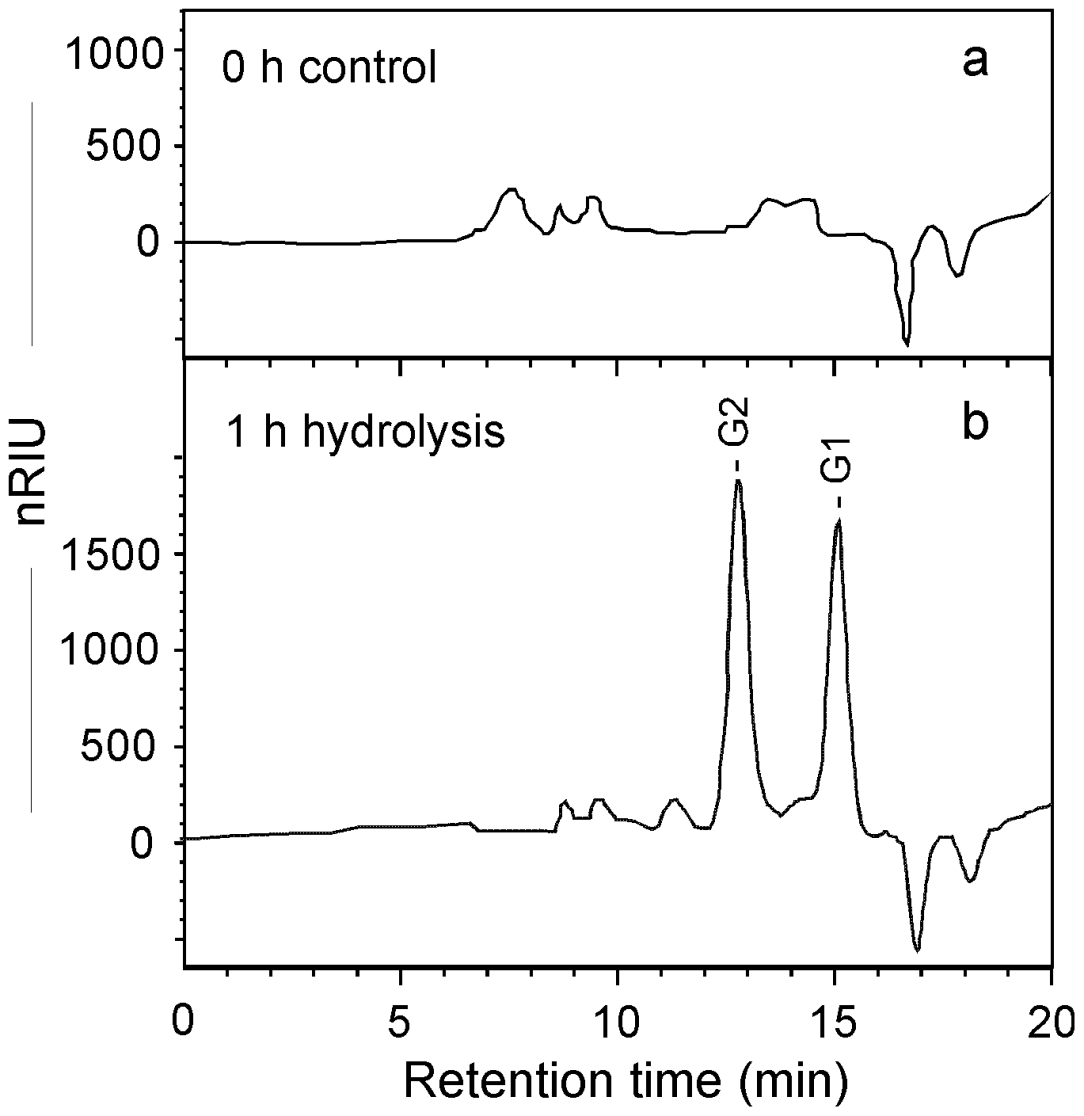
HPLC analysis of sugars released in hydrolysis of CMC by *M. circinelloides* extracellular enzymes. (**a**) 0 hour control; (**b**) 1 h hydrolysis. G1, glucose; G2, cellobiose.

To further investigate the cellulase activities of this strain, the extracellular crude enzyme solution was incubated with CMC or Avicel cellulose for 1 h. The profile of sugars liberated in the hydrolysis of CMC using crude extracellular enzymes from *M. circinelloides* cultures is shown in Figure 4b. The release of reducing sugars (including both cellobiose and glucose) from CMC indicated that *M. circinelloides* has clear hydrolytic capability for the β-1,4-glucosidic bonds in CMC. Again, this result is consistent with the observation of *M. circinelloides* growth on CMC plate. For the hydrolysis of Avicel by crude extracellular enzymes from *M. circinelloides* cultures, cellobiose was not detected, suggesting its ability to hydrolyze crystalline cellulose is limited.

### β-D-glucosidase activity and utilization of cellobiose

As described above, the genome analysis predicted two extracellular and one intracellular BGL; among them, BGL of ID 38405 was identified in the secretome. Here, we further tested the BGL enzymatic activity both of the supernatant as the crude enzyme and of the concentrated supernatant. The results indicated that both of them can convert cellobiose to glucose.

We also compared the strain's ability to utilize glucose *vs.* cellobiose for fungal mass growth, and the result is shown in Table 5. The results demonstrate that the medium with cellobiose as sole carbon source allowed the fungus to produce 50% (i.e. 2.09/4.15) of the cell mass (unit: g L^−1^ dry cell weight) that was produced on the medium with glucose as sole carbon source. It is noteworthy that the fungal mass yields with these two carbon sources are comparable, with a range of 0.20–0.23 g fungal mass yield per g sugar consumed.

**Table 5:** Growth of *M. circinelloides* on mimeral medium containing glucose *vs.* cellobiose as sole carbon source.

Substrate	DCW	Sugar consumed	Fungal mass yield[2]
	(g L^−1^)	(g L^−1^)	(g g^−1^ sugar)
Glucose	4.15	20.4	0.20
Cellobiose	2.09	8.9	0.23
No sugar addition (control)[1]	0.35	-	-

Data presented are the average values from duplicate experiments of 7 d culture.

**Notes:**

[1]. The no sugar addition (control) is the basal medium that contained yeast extract, which has low content of carbon that can support a minimal growth of fungus and explain the listed 0.35 g L^−1^ DCW (dry cell weight) in this table.

[2]. Fungal mass yield: fungal cell mass dry weight (g) produced per gram sugar consumed.

In summary, the data obtained so far indicates that the studied strain has the enzymatic activities of endoglucanase (in that it degrades CMC) and BGL (in that it degrades cellobiose), whereas it likely lacks the enzymatic activity of CBH1, which is vital for degrading crystalline cellulose.

### *Mucor* utilization of unsaccharified and pre-saccharified cellulosic substrates

In addition to evaluating the extracellular cellulolytic capacity, we also investigated both growth and lipid production by *M. circinelloides* on glucose, on unsaccharified and pre-saccharified Avicel cellulose, and on unsaccharified and pre-saccharified DAPCS, respectively. Among these experiments, the culturing of *M. circinelloides* on pre-saccharified Avicel cellulose and pre-saccharified DAPCS constitutes examination of the use of *M. circinelloides* in a SHF process.

**Figure 5:**
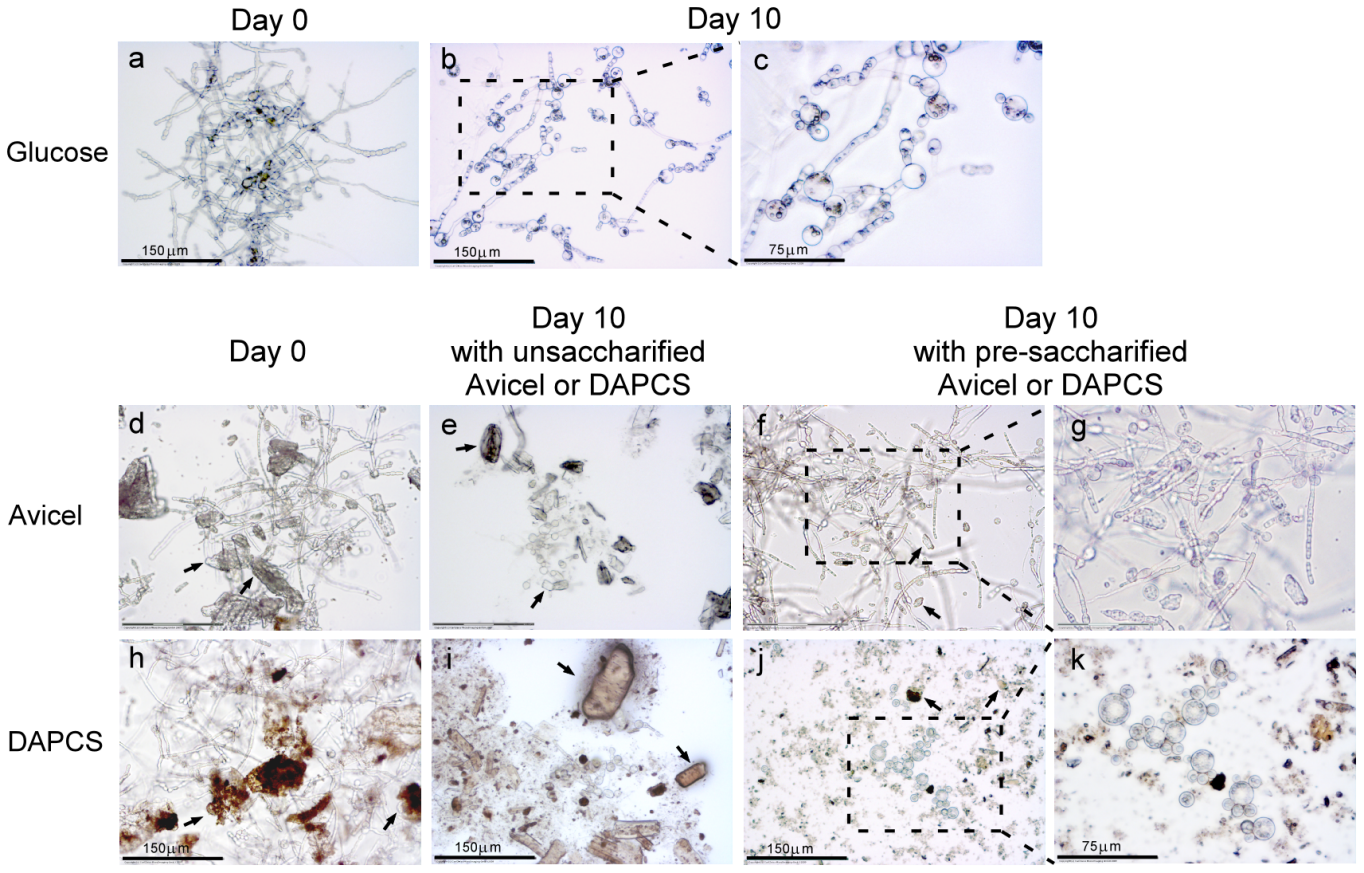
Morphological comparison of *M. circinelloides* grown on different substrates. The strain was cultured on medium containing glucose (a–c), unsaccharified and pre-saccharified Avicel (d–g), and unsaccharified and pre-saccharified DAPCS (dilute-acid pretreated corn stover) (h–k), respectively. In d–f, the arrow signs indicate the granules of Avicel powder particles, while in h–j, the arrow signs indicate the DAPCS particles. The scale bar represents 150 µm for a–f and h–j, and 75 µm for g and k.

Microscopic observation of the fungal growth was carried out using laser dissection microscopy (Carl Zeiss AG, Germany). 3% (wt/v) glucose as carbon source was added to the lipid production (LP) medium for fungal growth and lipid production. There was no change of morphology when the fungus was transferred from PD preculture into glucose medium, the yeast remaining in clusters of filaments at Day 0 (Figure 5a). After 10 d of culturing, the broth was occupied with dispersed, branched mycelia (Figure 5 b–c), in which the deep colored enclosures appeared to be lipid droplets (Figure 5c). FAME analysis showed a lipid content of 22.7% of (w/w of dry weight of cells). The FAME profiles are described in Table 6.

**Table 6:** Fatty acid profile of *M. circinelloides* in lipid production medium with glucose and pre-saccharified Avicel as C (carbon) sources.

	Fatty acid composition (%)																
C sources	C10	C12	C14	C16:3	C16:4	C16:2	C16:1n9	C16:0	C18:2	C18:1n9	C18:3	C18:0	C20	C22:1n9	C22	C24	Total fatty acids
**Glucose**	0.5±0	0.7±0	**3.4±0**	0.5±0	0.5±0	**0.6±0**	**2.9±0**	**29.6±0**	**15.5±0.4**	38.4±0.6	0.6±0	4.6±0.2	0.5±0	0.3±0	0.6±0	0.9±0	100
**Avicel**	ND	ND	**6.9±0.3** **	ND	ND	**2.8±0.3** **	**7.1±0.3** **	**22.5±0.3** *	**11.9±0.4** *	36.9±1.1	2.6±0.5	6.6±0.2	ND	ND	ND	3.6±0.4	100

The total FAME (% w/w of DW) was described in the text of Results and Discussion section. Data shown are the mean from three replicate measurements ± standard deviation of the mean (SEM). Student's t-test was used to evaluate the statistical significance of the observed differences between the proportion of a given fatty acid in the mixtures produced from two different carbon sources.

* indicates statistical significance of p<0.05;

** indicates statistical significance of p<0.01.

Compared with glucose-grown *M. circinelloides*, the most prominent feature of the unsaccharified Avicel- or unsaccharified DAPCS-grown *M. circinelloides* is that at Day 10, little growth of cells was observed, and most of them were scattered cells in yeast form (Figure 5 e and i). However, with pre-saccharified Avicel or DAPCS as carbon substrates, *M. circinelloides* can support fungal growth and produce lipid-like droplets (Figure 5 f–g and j–k). FAME analysis of cells grown on pre-saccharified Avicel revealed a lipid content equal to 4.5% of the dry weight of the mixture of cells and residual Avicel. Because the calculated FAME content is based on the dry weight of the *Mucor* cell-Avicel residue mixture, the lipid content unavoidably appears to be much lower than it would be on a pure cell basis. In the literature, this has not been an issue since soluble substrates like glucose do not become part of the harvested pellet; in contrast, the solid substrates – the Avicel and DAPCS in this case – complicate the cell harvesting. Further study and method-development are needed to address this issue.

It is noteworthy, however, that a comparison at Day 10 between *Mucor* grown on pre-saccharified Avicel and on pre-saccharified DAPCS revealed that the former was dominated by filamentous fungal forms (Figure 5 f–g) and the latter by yeast-like forms, suggesting that some extent of inhibition may affect DAPCS-grown *Mucor* (Figure 5 j–k). Although the mechanism of this morphogenesis transition is not clear yet, it has been observed that the biosynthesis of unsaturated fatty acids and sterols are inhibited under yeast development conditions [33]; in the present circumstance, the observed yeast-like morphogenesis may be caused by the toxicity of DAPCS-related components.

In summary, these results suggest that although the unmodified *M. circinelloides* itself cannot utilize Avicel or DAPCS directly, a complementary approach of introducing cellulase genes into *Mucor* is likely to make it capable of utilizing cellulosic materials.

### Impact of pre-saccharified cellulolytic substrate on fatty acid profiling

The FAME profiles of glucose- *vs.* pre-saccharified Avicel-grown *Mucor* cells are worth some discussion (Table 6). The FAME profile in both types of cells shows major fatty acids being C18:1n9, C16:0, C18:2, C16:1, C18:0 and C14 (in order of prominence) with a relatively minor contribution from C24 (Table 6). Overall, such a profile is consistent with the literature for the closely related strains *C. echinulata* and *M. isabellina* [34], [35], with the exception of the C14 fatty acids. The profile actually varied between these two substrates and could indicate some significant reshuffling in the lipids based on carbon sources. Intriguingly, the pre-saccharified Avicel substrate had a higher C14 fatty acid content, which has an interesting practical implication as shorter fatty acid chains are more ideal for jet fuel characteristics [36]. This substrate- specific shift in FAME profile may warrant further investigation.

### Proof of concept for an envisioned consolidated bioprocessing by supplementation with cellobiohydrolase I

Our above genomic and secretomic search for cellulases in *M. circinelloides* showed that it has endoglucanases and BGLs, and their activities were also demonstrated. These together prompted us to propose that *M. circinelloides* can be developed as a microorganism capable of converting biomass directly to lipids.

**Figure 6:**
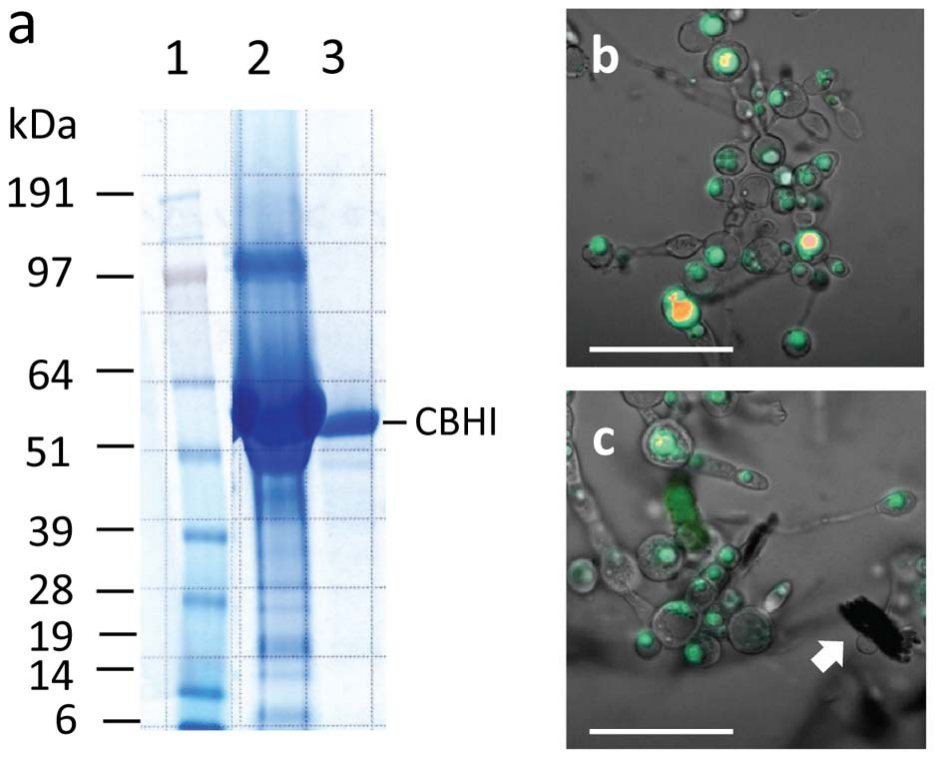
SDS–PAGE analysis of supplemental CBHI and Nile red staining of lipids in *M. circinelloide*. (**a**) SDS–PAGE of the original and purified commercial *T. longibrachiatum* CBHI. Lane 1, molecular weight standards; lane 2, CBHI from the vial of the supplier with a loading amount of 10 µL; lane 3, CBHI after further hydrophobic interaction chromatography (HIC) and gel filtration purification, with a loading amount of 10 µL. (**b**) Nile red staining of *M. circinelloide* grown on glucose medium. (**c**) Nile red staining of *M. circinelloide* grown on Avicel medium supplemented with the above purified CBHI. Both (**b**) and (**c**) are combined images in which the fluorescence from the Nile Red is superimposed on the white light image. In (**c**), the arrow sign indicates the granular residue of Avicel powder particles. The scale bar represents 60 µm for the images.

To test the feasibility of the above proposal, we cultured *M. circinelloides* in the medium with Avicel supplemented with CBHI as the sole carbon source, comparing its performance with that of the organism on an otherwise identical medium with glucose as carbon source. PAGE analysis shown in Figure 6a confirms the purity of the CBHI used in the experiment. *Mucor* grown in both medium with glucose or with Avicel-CBHI revealed significant numbers of lipid bodies, which were visualized by fluorescence microscopy of the cells after staining with Nile Red (Figure 6 b–c). To obtain an accurate comparison of lipid accumulation, the cell or cell-Avicel residue pellets were freeze-dried and subjected to analyses for their FAME yield.

The fungal mass and lipid production are shown in Table 7. The results demonstrated that with CBHI as a supplement, *Mucor* can grow on Avicel and consumed more than one-third of the original Avicel content, producing 0.676 g FAME per liter culture, which is nearly one third of the total FAME produced in glucose medium (2.095 g FAME per liter; Table 7). It is noteworthy that the FAME yield rate (sugar-consumption basis) with Avicel-CBHI was 56.62 mg FAME per g glucose consumed, which was 80% of the FAME yield with glucose medium.

**Table 7:** Growth of *M. circinelloides* on mineral medium containing glucose *vs.* Avicel supplemented with CBHI enzyme as sole carbon sources.

Carbon	Pellet weight	Total FAME	% Total FAME - pellet dry weight basis		Carbon balance			FAME yield[4]
(in setup medium)		(in whole pellet)	Mean	Std Dev	Glu in Supernat	Glucan in Avicel residue[3]	Sugar consumed	
	(g L^−1^)	(g L^−1^)			(Glu g L^−1^)	(Glu g L^−1^)	(Glu g L^−1^)	(mg g^−1^ Glu)
(1) No-C control[1]	0.38	0.004	1.16%	0.02%	0.00	-		
(2) Glu 30 g/L	10.78	2.095	19.44%	0.18%	0.06	-	29.94	69.99
(3) Avicel 27 g/L+CBH1[2]	20.33	0.676	3.32%	0.05%	0.04	18.02	11.94	56.62

Data presented are the average values from duplicate experiments. CBHI, cellobiohydrolase I; Glu, glucose; Supernat, supernatant.

**Note:**

[1] The no sugar control basal medium contained yeast extract, which has low content of carbon that can support a minimal growth of fungus.

[2] The pellet contained the fungal cell mass for the no-C (carbon) control and glucose media, and cell mass plus residual Avicel for the Avicel-CBHI medium.

[3] Glucan in Avicel residue is presented as glucose equivalent unit.

[4] FAME yield: amount (mg) of FAME produced per gram glucose consumed.

## Conclusion

The current study constitutes, to the best of our knowledge, the first comprehensive analyses (as illustrated in Figure 1) of the cellulolytic capacity of *M. circinelloides*. The genomic and secretomic data, along with the observed enzymatic activity and its capability of utilizing CMC and cellobiose, suggest that *M. circinelloides* possesses a significant cellulolytic system that includes endoglucanases and BGL but not the exoglucanases. The demonstrated efficiency of *M. circinelloides* in utilizing pre-saccharified cellulose and DAPCS, and especially Avicel supplemented with CBHI enzymes, for fungal growth and lipid production supports the feasibility of our proposal, which is aimed at developing *M. circinelloides* as a CBP strain capable of converting biomass directly to biofuels. Our data also suggest that its cellulolytic capacity can be further increased by enhancing the protein expression level of the major BGLs, as currently the mass of fungal cells growing on cellobiose substrate was half of that growing on glucose.

## Supporting Information

Table S1 **List of 259 GHs, 126 CEs and other CAZymes based on the genome-wide CAZy family protein analysis of** ***M. circinelloides*** **.**
(PDF)

Table S2 **List of peptide sequences and proteins identified in the bands of A0–A9 and B0–B8 from concentrator- and ethanol-prepared protein samples, respectively.**
(PDF)
