# Supplementary material for: Genomic, Proteomic, and Biochemical Analyses of Oleaginous Mucor circinelloides: Evaluating Its Capability in Utilizing Cellulolytic Substrates for Lipid Production
Source: PLoS One. 2013 Sep 4;8(9):e71068. doi: 10.1371/journal.pone.0071068 (PMC3762813; doi:10.1371/journal.pone.0071068)
Supplement: Table S1 — List of 259 GHs, 126 CEs and other CAZymes based on the genome-wide CAZy family protein analysis of M. circinelloides . (PDF) [file pone.0071068.s001.pdf]

Manuscript title: Genomic, proteomic, and biochemical analyses of oleaginous *Mucor circinelloides* : Evaluating its capability in utilizing cellulolytic substrates for lipid production

Supplemental Table S1. List of 259 GHs, 126 CEs and other CAZymes based on the genome-wide Cazy family protein analysis of *M. circinelloides.*

Notes: The proteins are sorted in the alphabetical order of CAZyme proteins.

Index	Mucor Protein ID	CAZy Families	Similarity matched protein and source	
			GenBank accession no.	Organism
1	128301	CBM1	ADT70774.1	Dichomera saubinetii CBS 990.70
2	165351	CBM12	ACX42259.1	Paenibacillus dendritiformis T451
3	76500	CBM12|CBM32	ABX04461.1	Herpetosiphon aurantiacus ATCC 23779
4	124855	CBM12|CBM32	ABX04461.1	Herpetosiphon aurantiacus ATCC 23779
5	134035	CBM12|CBM32	ABX04461.1	Herpetosiphon aurantiacus ATCC 23779
6	154711	CBM12|CBM32	ABX04461.1	Herpetosiphon aurantiacus ATCC 23779
7	154804	CBM12|CBM32	ABX04461.1	Herpetosiphon aurantiacus ATCC 23779
8	80145	CBM12|CBM32	ABX04461.1	Herpetosiphon aurantiacus ATCC 23779
9	154969	CBM12|CBM32	ABX04461.1	Herpetosiphon aurantiacus ATCC 23779
10	134047	CBM12|CBM32	ABX04461.1	Herpetosiphon aurantiacus ATCC 23779
11	148187	CBM12|CBM32	ABX04461.1	Herpetosiphon aurantiacus ATCC 23779
12	37441	CBM12|CBM32	ABX04461.1	Herpetosiphon aurantiacus ATCC 23779
13	155073	CBM12|CBM32	ABX04461.1	Herpetosiphon aurantiacus ATCC 23779
14	150820	CBM12|CBM32	ABX04461.1	Herpetosiphon aurantiacus ATCC 23779
15	104660	CBM12|CBM32	ABX04461.1	Herpetosiphon aurantiacus ATCC 23779
16	174255	CBM13	CAJ58949.1	Frankia alni ACN14a
17	143123	CBM13	CAJ61616.1	Frankia alni ACN14a
18	143745	CBM13	CAJ63931.1	Frankia alni ACN14a
19	156405	CBM13	ADO75815.1	Stigmatella aurantiaca DW4/3-1
20	156763	CBM13	CAJ63931.1	Frankia alni ACN14a
21	157185	CBM13	ADO75815.1	Stigmatella aurantiaca DW4/3-1
22	185951	CBM13	CAJ61616.1	Frankia alni ACN14a
23	153890	CBM13	CAJ63931.1	Frankia alni ACN14a
24	121913	CBM13	CAJ63931.1	Frankia alni ACN14a
25	137539	CBM13	CAJ63931.1	Frankia alni ACN14a
26	156689	CBM13	ADN17604.1	Cyanothece sp. PCC 7822
27	141963	CBM13	CAJ63931.1	Frankia alni ACN14a
28	16210	CBM13	CAJ63931.1	Frankia alni ACN14a
29	78822	CBM13	CAJ58949.1	Frankia alni ACN14a
30	88941	CBM13	CAJ61616.1	Frankia alni ACN14a
31	31506	CBM13	CAJ61616.1	Frankia alni ACN14a
32	73572	CBM13	CAJ63931.1	Frankia alni ACN14a
33	87139	CBM13	CAJ63931.1	Frankia alni ACN14a
34	187297	CBM13	CAJ61616.1	Frankia alni ACN14a
35	22248	CBM13	ADO75815.1	Stigmatella aurantiaca DW4/3-1
36	181703	CBM13	CAJ58949.1	Frankia alni ACN14a

37	136605	CBM13	CAJ58949.1	Frankia alni ACN14a
38	122109	CBM13	CAJ63931.1	Frankia alni ACN14a
39	35714	CBM13	CAJ63931.1	Frankia alni ACN14a
40	130722	CBM13	ADO75815.1	Stigmatella aurantiaca DW4/3-1
41	171806	CBM13	CAJ58949.1	Frankia alni ACN14a
42	137550	CBM13	CAJ58949.1	Frankia alni ACN14a
43	95896	CBM13	CAJ58949.1	Frankia alni ACN14a
44	120703	CBM13	ADO75815.1	Stigmatella aurantiaca DW4/3-1
45	189295	CBM13	CAJ61616.1	Frankia alni ACN14a
46	90308	CBM13	CAJ61616.1	Frankia alni ACN14a
47	132239	CBM13	CAJ63931.1	Frankia alni ACN14a
48	130327	CBM13	CAJ58949.1	Frankia alni ACN14a
49	130592	CBM13	CAJ58949.1	Frankia alni ACN14a
50	47751	CBM13	CAJ58949.1	Frankia alni ACN14a
51	36903	CBM13	CAJ63931.1	Frankia alni ACN14a
52	143652	CBM13	CAJ63931.1	Frankia alni ACN14a
53	153694	CBM13	CAJ63931.1	Frankia alni ACN14a
54	130262	CBM13	CAJ63931.1	Frankia alni ACN14a
55	140040	CBM13	CAJ63931.1	Frankia alni ACN14a
56	137721	CBM13	CAJ63931.1	Frankia alni ACN14a
57	156428	CBM13	ADN17604.1	Cyanothece sp. PCC 7822
58	154274	CBM13	ADO75815.1	Stigmatella aurantiaca DW4/3-1
59	127302	CBM13	CAJ63931.1	Frankia alni ACN14a
60	112109	CBM13	CAJ58949.1	Frankia alni ACN14a
61	146655	CBM13	CAJ58949.1	Frankia alni ACN14a
62	42753	CBM13	CAJ58949.1	Frankia alni ACN14a
63	154977	CBM13	CAJ63931.1	Frankia alni ACN14a
64	136417	CBM13	CAJ58949.1	Frankia alni ACN14a
65	146494	CBM13	CAJ58949.1	Frankia alni ACN14a
66	189067	CBM13	CAJ61616.1	Frankia alni ACN14a
67	155277	CBM13	CAJ63931.1	Frankia alni ACN14a
68	114701	CBM13	ADN17604.1	Cyanothece sp. PCC 7822
69	129018	CBM13	CAJ58949.1	Frankia alni ACN14a
70	129018	CBM13	CAJ63931.1	Frankia alni ACN14a
71	139389	CBM13	CAJ63931.1	Frankia alni ACN14a
72	146914	CBM13	CAJ58949.1	Frankia alni ACN14a
73	119278	CBM13	CAJ63931.1	Frankia alni ACN14a
74	171231	CBM13	CAJ63931.1	Frankia alni ACN14a
75	43544	CBM13	ADO75815.1	Stigmatella aurantiaca DW4/3-1
76	76134	CBM13	CAJ61616.1	Frankia alni ACN14a
77	146440	CBM13	CAJ61616.1	Frankia alni ACN14a
78	75120	CBM13	CAJ63931.1	Frankia alni ACN14a
79	109224	CBM13	CAJ63931.1	Frankia alni ACN14a
80	72528	CBM13	NP_733639.1	Streptomyces coelicolor A3(2)
81	177367	CBM13	CAJ61616.1	Frankia alni ACN14a
82	186853	CBM13	CAJ58949.1	Frankia alni ACN14a
83	157589	CBM13	CAJ58949.1	Frankia alni ACN14a

84	132375	CBM13	CAJ58949.1	Frankia alni ACN14a
85	118716	CBM13	CAJ63931.1	Frankia alni ACN14a
86	87923	CBM13	CAJ63931.1	Frankia alni ACN14a
87	155258	CBM13	NP_733639.1	Streptomyces coelicolor A3(2)
88	134587	CBM13	CAJ63931.1	Frankia alni ACN14a
89	157302	CBM13	CAJ58949.1	Frankia alni ACN14a
90	46417	CBM13	CAJ58949.1	Frankia alni ACN14a
91	153211	CBM13	CAJ58949.1	Frankia alni ACN14a
92	153211	CBM13	CAJ63931.1	Frankia alni ACN14a
93	135438	CBM13	CAJ63931.1	Frankia alni ACN14a
94	121008	CBM13	CAJ58949.1	Frankia alni ACN14a
95	139510	CBM13	CAJ63931.1	Frankia alni ACN14a
96	122121	CBM13	CAJ63931.1	Frankia alni ACN14a
97	128468	CBM13	CAJ63931.1	Frankia alni ACN14a
98	104413	CBM13	CAJ58949.1	Frankia alni ACN14a
99	104413	CBM13	CAJ63931.1	Frankia alni ACN14a
100	118814	CBM13	CAJ63931.1	Frankia alni ACN14a
101	119441	CBM14	ABI95429.1	Lucilia cuprina
102	119441	CBM14	ADD18365.1	Glossina morsitans morsitans
103	185052	CBM14	ABI95429.1	Lucilia cuprina
104	186155	CBM14	ABI95429.1	Lucilia cuprina
105	12145	CBM14	ADD18365.1	Glossina morsitans morsitans
106	164292	CBM18	CBX91631.1	Leptosphaeria maculans v23.1.3
107	155688	CBM18	CBX91631.1	Leptosphaeria maculans v23.1.3
108	157300	CBM18	CAD89077.1	Botryotinia fuckeliana
109	156933	CBM18	CBX91631.1	Leptosphaeria maculans v23.1.3
110	163810	CBM18	CAD89674.1	Botryotinia fuckeliana SAS56
111	142925	CBM18	CBX91631.1	Leptosphaeria maculans v23.1.3
112	157301	CBM18	CAD89674.1	Botryotinia fuckeliana SAS56
113	157301	CBM18	CAD89077.1	Botryotinia fuckeliana
114	187130	CBM18	CBX91631.1	Leptosphaeria maculans v23.1.3
115	19138	CBM18	CBX91631.1	Leptosphaeria maculans v23.1.3
116	139507	CBM2	ADJ44991.1	Amycolatopsis mediterranei U32
117	164694	CBM2	ADJ44991.1	Amycolatopsis mediterranei U32
118	130565	CBM2	ADJ44991.1	Amycolatopsis mediterranei U32
119	13818	CBM2	ADJ44991.1	Amycolatopsis mediterranei U32
120	156730	CBM2	ADJ44991.1	Amycolatopsis mediterranei U32
121	146462	CBM2	ACU38029.1	Actinosynnema mirum DSM 43827
122	38081	CBM2	ADL49113.1	Micromonospora aurantiaca ATCC 27029
123	38081	CBM2	ADU08409.1	Micromonospora sp. L5
124	132262	CBM2	ADJ44991.1	Amycolatopsis mediterranei U32
125	41460	CBM2	ADJ44991.1	Amycolatopsis mediterranei U32
126	135599	CBM2	ADJ44991.1	Amycolatopsis mediterranei U32
127	121377	CBM2	ADJ47377.1	Amycolatopsis mediterranei U32
128	121377	CBM2	ACU38029.1	Actinosynnema mirum DSM 43827
129	121280	CBM2	ADJ44991.1	Amycolatopsis mediterranei U32
130	157459	CBM2	ADJ44991.1	Amycolatopsis mediterranei U32

131	131695	CBM2	ACU38029.1	Actinosynnema mirum DSM 43827
132	167065	CBM2	ACU38029.1	Actinosynnema mirum DSM 43827
133	43146	CBM2	ADJ44991.1	Amycolatopsis mediterranei U32
134	43377	CBM2	ADJ44991.1	Amycolatopsis mediterranei U32
135	108853	CBM2	ADL49113.1	Micromonospora aurantiaca ATCC 27029
136	108853	CBM2	ADU08409.1	Micromonospora sp. L5
137	152497	CBM2	ADJ44991.1	Amycolatopsis mediterranei U32
138	109327	CBM2	ADL49113.1	Micromonospora aurantiaca ATCC 27029
139	109327	CBM2	ADU08409.1	Micromonospora sp. L5
140	155167	CBM2	ADJ44991.1	Amycolatopsis mediterranei U32
141	148701	CBM2	ADJ44990.1	Amycolatopsis mediterranei U32
142	149405	CBM2	ACU38029.1	Actinosynnema mirum DSM 43827
143	132261	CBM2	ADJ44991.1	Amycolatopsis mediterranei U32
144	45426	CBM2	ADJ44991.1	Amycolatopsis mediterranei U32
145	44416	CBM2	ADJ44991.1	Amycolatopsis mediterranei U32
146	134469	CBM2	ADJ44991.1	Amycolatopsis mediterranei U32
147	129678	CBM2	ADJ44991.1	Amycolatopsis mediterranei U32
148	143038	CBM2	ADJ47377.1	Amycolatopsis mediterranei U32
149	132101	CBM2	ADJ44991.1	Amycolatopsis mediterranei U32
150	117701	CBM2	ACU38029.1	Actinosynnema mirum DSM 43827
151	110137	CBM2	ADL49113.1	Micromonospora aurantiaca ATCC 27029
152	110137	CBM2	ADU08409.1	Micromonospora sp. L5
153	148343	CBM2	ADJ44991.1	Amycolatopsis mediterranei U32
154	31739	CBM2	ACU38029.1	Actinosynnema mirum DSM 43827
155	148930	CBM2	ADL49113.1	Micromonospora aurantiaca ATCC 27029
156	148930	CBM2	ADU08409.1	Micromonospora sp. L5
157	113165	CBM2	ADJ44991.1	Amycolatopsis mediterranei U32
158	157497	CBM2	ADJ44991.1	Amycolatopsis mediterranei U32
159	132767	CBM2	ACU38029.1	Actinosynnema mirum DSM 43827
160	121205	CBM2	ACU38029.1	Actinosynnema mirum DSM 43827
161	147728	CBM2	ADJ44991.1	Amycolatopsis mediterranei U32
162	133817	CBM2	ABM33954.1	Acidovorax avenae subsp. citrulli AAC00-1
163	121051	CBM2	ADJ44991.1	Amycolatopsis mediterranei U32
164	154806	CBM2	ADJ44991.1	Amycolatopsis mediterranei U32
165	167661	CBM20	AAI08539.1	Xenopus laevis
166	144012	CBM20	NP_942074.1	Rattus norvegicus
167	144012	CBM20	AAI08539.1	Xenopus laevis
168	113939	CBM20	ACD23569.1	Clostridium botulinum B str. Eklund 17B
169	135169	CBM20|GH15	EAA64118.1	Aspergillus nidulans FGSC A4
170	79895	CBM21	AAW44639.1	Cryptococcus neoformans var. neoformans JEC21
171	113493	CBM21	CAP80870.1	Penicillium chrysogenum Wisconsin 54-1255
172	182252	CBM21	CAK37632.1	Aspergillus niger CBS 513.88
173	157380	CBM21	CAR28223.1	Zygosaccharomyces rouxii CBS 732
174	110400	CBM21	CBX97277.1	Leptosphaeria maculans v23.1.3
175	128399	CBM32	ACU76193.1	Catenulispora acidiphila DSM 44928
176	163388	CBM32	ADO69217.1	Stigmatella aurantiaca DW4/3-1
177	75579	CBM32	ADO69217.1	Stigmatella aurantiaca DW4/3-1

178	155429	CBM32	ADO69217.1	Stigmatella aurantiaca DW4/3-1
179	18304	CBM33	NP_258300.1	Spodoptera litura NPV G2
180	157563	CBM33	NP_258300.1	Spodoptera litura NPV G2
181	111742	CBM33	NP_258300.1	Spodoptera litura NPV G2
182	156286	CBM33	NP_258300.1	Spodoptera litura NPV G2
183	156420	CBM33	NP_258300.1	Spodoptera litura NPV G2
184	156757	CBM33	NP_258300.1	Spodoptera litura NPV G2
185	181198	CBM33	NP_258300.1	Spodoptera litura NPV G2
186	19465	CBM33	NP_258300.1	Spodoptera litura NPV G2
187	107469	CBM42|CE2	ADO71607.1	Stigmatella aurantiaca DW4/3-1
188	35482	CBM47	ABJ81631.1	Solibacter usitatus Ellin6076
189	123569	CBM48	ACY19019.1	Haliangium ochraceum DSM 14365
190	32400	CBM48	ACY19019.1	Haliangium ochraceum DSM 14365
191	121280	CBM48	ACY19019.1	Haliangium ochraceum DSM 14365
192	32392	CBM48	ABO98260.1	Ostreococcus lucimarinus CCE9901
193	134872	CBM48	XP_503921.1	Yarrowia lipolytica W29
194	156988	CBM48	NP_011307.1	Saccharomyces cerevisiae S288C
195	146132	CBM48	ABO98260.1	Ostreococcus lucimarinus CCE9901
196	147215	CBM48	ACY19019.1	Haliangium ochraceum DSM 14365
197	74093	CBM48	ACY19019.1	Haliangium ochraceum DSM 14365
198	163802	CBM48	XP_504985.1	Yarrowia lipolytica W29
199	163802	CBM48	CAG77792.1	Yarrowia lipolytica CLIB122 CLIB99
200	28137	CBM48	ACY19019.1	Haliangium ochraceum DSM 14365
201	16644	CBM48	ACY19019.1	Haliangium ochraceum DSM 14365
202	156822	CBM48|GH13	ACI49189.1	Caenorhabditis sp. PS1010
203	179277	CBM48|GH13	ACI49189.1	Caenorhabditis sp. PS1010
204	110916	CBM48|GH13	ACI49189.1	Caenorhabditis sp. PS1010
205	50331	CBM48|GH13	ACI49189.1	Caenorhabditis sp. PS1010
206	114707	CBM48|GH13	ACI49189.1	Caenorhabditis sp. PS1010
207	151269	CBM48|GH13	ACI49189.1	Caenorhabditis sp. PS1010
208	187168	CBM48|GH13	CBX90546.1	Leptosphaeria maculans v23.1.3
209	120185	CBM48|GH13	ACI49189.1	Caenorhabditis sp. PS1010
210	155478	CBM48|GH13	ACI49189.1	Caenorhabditis sp. PS1010
211	114956	CBM48|GH13	ACI49189.1	Caenorhabditis sp. PS1010
212	143079	CBM48|GH13	ACI49189.1	Caenorhabditis sp. PS1010
213	155154	CBM48|GH13	ACI49189.1	Caenorhabditis sp. PS1010
214	84685	CBM48|GH13	ACI49189.1	Caenorhabditis sp. PS1010
215	130191	CBM48|GH13	ACI49189.1	Caenorhabditis sp. PS1010
216	78912	CBM5	ACV48841.1	Halomicrobium mukohataei DSM 12286
217	105526	CBM50	CBI17583.3	Vitis vinifera
218	157069	CBM50	ABQ59610.1	Glycine max
219	157069	CBM50	EEC71457.1	Oryza sativa Indica Group
220	157069	CBM50	EEE55360.1	Oryza sativa Japonica Group
221	80409	CBM50	NP_175606.2	Arabidopsis thaliana
222	130571	CBM50	CBI36053.3	Vitis vinifera
223	106028	CBM50	ADK79942.1	Spirochaeta smaragdinae DSM 11293
224	114869	CBM50	NP_175606.2	Arabidopsis thaliana

225	114869	CBM50	BAI79272.1	Lotus japonicus
226	127721	CBM50	ACS99118.1	Paenibacillus sp. JDR-2
227	127721	CBM50	ACD21945.1	Clostridium botulinum B str. Eklund 17B
228	166257	CBM51	ABF44103.1	Deinococcus geothermalis DSM 11300
229	117745	CBM57	BAJ53166.1	Jatropha curcas Palawan
230	135298	CBM57	BAJ53166.1	Jatropha curcas Palawan
231	23274	CBM57	BAJ53166.1	Jatropha curcas Palawan
232	166440	CBM57	BAJ53166.1	Jatropha curcas Palawan
233	106678	CBM57	BAJ53166.1	Jatropha curcas Palawan
234	145801	CBM57	BAJ53166.1	Jatropha curcas Palawan
235	166534	CBM57	ACZ43662.1	Thermobaculum terrenum ATCC BAA-798
236	147534	CBM57	BAJ53166.1	Jatropha curcas Palawan
237	39384	CBM57	BAJ53166.1	Jatropha curcas Palawan
238	147820	CBM6	CAM03105.1	Saccharopolyspora erythraea NRRL 2338
239	156763	CBM6	CAM03105.1	Saccharopolyspora erythraea NRRL 2338
240	180465	CBM6	CAM03105.1	Saccharopolyspora erythraea NRRL 2338
241	157625	CBM6	CAM03105.1	Saccharopolyspora erythraea NRRL 2338
242	153277	CBM6	CAM03105.1	Saccharopolyspora erythraea NRRL 2338
243	15556	CBM6	CAM03105.1	Saccharopolyspora erythraea NRRL 2338
244	155028	CBM6	CAM03105.1	Saccharopolyspora erythraea NRRL 2338
245	159850	CBM6	CAM03105.1	Saccharopolyspora erythraea NRRL 2338
246	74597	CBM6	CAM03105.1	Saccharopolyspora erythraea NRRL 2338
247	33029	CBM6	CAM03105.1	Saccharopolyspora erythraea NRRL 2338
248	88295	CBM6	CAM03105.1	Saccharopolyspora erythraea NRRL 2338
249	155376	CBM6	CAM03105.1	Saccharopolyspora erythraea NRRL 2338
250	155455	CBM6	CAM03105.1	Saccharopolyspora erythraea NRRL 2338
251	121993	CBM6	CAM03105.1	Saccharopolyspora erythraea NRRL 2338
252	142968	CBM6	CAM03105.1	Saccharopolyspora erythraea NRRL 2338
253	138180	CBM6	CAM03105.1	Saccharopolyspora erythraea NRRL 2338
254	137550	CBM6	CAM03105.1	Saccharopolyspora erythraea NRRL 2338
255	11770	CBM6	CAM03105.1	Saccharopolyspora erythraea NRRL 2338
256	151782	CBM6	CAM03105.1	Saccharopolyspora erythraea NRRL 2338
257	132379	CBM6	CAM03105.1	Saccharopolyspora erythraea NRRL 2338
258	141754	CBM6	CAM03105.1	Saccharopolyspora erythraea NRRL 2338
259	154281	CBM6	CAM03105.1	Saccharopolyspora erythraea NRRL 2338
260	134876	CBM6	CAM03105.1	Saccharopolyspora erythraea NRRL 2338
261	156022	CBM6	CAM03105.1	Saccharopolyspora erythraea NRRL 2338
262	139960	CBM6	CAM03105.1	Saccharopolyspora erythraea NRRL 2338
263	120294	CBM6	ABP54505.1	Salinispora tropica CNB-440
264	119060	CBM6	CAM03105.1	Saccharopolyspora erythraea NRRL 2338
265	122503	CBM6	CAM03105.1	Saccharopolyspora erythraea NRRL 2338
266	124558	CBM6	CAM03105.1	Saccharopolyspora erythraea NRRL 2338
267	154881	CBM6	CAM03105.1	Saccharopolyspora erythraea NRRL 2338
268	175622	CBM6	CAM03105.1	Saccharopolyspora erythraea NRRL 2338
269	142601	CBM6	CAM03105.1	Saccharopolyspora erythraea NRRL 2338
270	17218	CBM6	CAM03105.1	Saccharopolyspora erythraea NRRL 2338
271	149791	CBM6	CAM03105.1	Saccharopolyspora erythraea NRRL 2338

272	118577	CBM6	CAM03105.1	Saccharopolyspora erythraea NRRL 2338
273	31420	CBM6	CAM03105.1	Saccharopolyspora erythraea NRRL 2338
274	142198	CBM6	CAM03105.1	Saccharopolyspora erythraea NRRL 2338
275	144517	CBM6	CAM03105.1	Saccharopolyspora erythraea NRRL 2338
276	132128	CBM6	CAM03105.1	Saccharopolyspora erythraea NRRL 2338
277	32379	CBM6	CAM03105.1	Saccharopolyspora erythraea NRRL 2338
278	120216	CBM6	CAM03105.1	Saccharopolyspora erythraea NRRL 2338
279	130028	CBM6	CAM03105.1	Saccharopolyspora erythraea NRRL 2338
280	132229	CBM6	CAM03105.1	Saccharopolyspora erythraea NRRL 2338
281	74552	CBM6	CAM03105.1	Saccharopolyspora erythraea NRRL 2338
282	144114	CBM6	CAM03105.1	Saccharopolyspora erythraea NRRL 2338
283	87831	CBM6	CAM03105.1	Saccharopolyspora erythraea NRRL 2338
284	157961	CBM6	CAM03105.1	Saccharopolyspora erythraea NRRL 2338
285	147729	CBM6	CAM03105.1	Saccharopolyspora erythraea NRRL 2338
286	148528	CBM6	CAM03105.1	Saccharopolyspora erythraea NRRL 2338
287	148732	CBM6	CAM03105.1	Saccharopolyspora erythraea NRRL 2338
288	34313	CBM6	CAM03105.1	Saccharopolyspora erythraea NRRL 2338
289	162827	CE1	CAI12228.1	Homo sapiens
290	152855	CE1	ACO62070.1	Micromonas sp. RCC299
291	151823	CE1	AAV54596.1	Sterkiella histriomuscorum
292	156685	CE1	AAV54596.1	Sterkiella histriomuscorum
293	155501	CE1	AAV54596.1	Sterkiella histriomuscorum
294	185587	CE10	NP_518560.1	Ralstonia solanacearum GMI1000
295	94368	CE10	NP_421101.1	Caulobacter crescentus CB15
296	167388	CE10	AAF70342.1	Psychrobacter sp. St1
297	147952	CE10	NP_593970.1	Schizosaccharomyces pombe 972h-
298	161809	CE10	NP_518560.1	Ralstonia solanacearum GMI1000
299	115752	CE10	NP_631192.1	Streptomyces coelicolor A3(2)
300	84104	CE10	NP_631192.1	Streptomyces coelicolor A3(2)
301	115264	CE10	NP_518560.1	Ralstonia solanacearum GMI1000
302	81713	CE10	NP_631192.1	Streptomyces coelicolor A3(2)
303	104772	CE10	NP_298542.1	Xylella fastidiosa 9a5c
304	117489	CE10	BAA10507.1	Synechocystis sp. PCC 6803
305	109092	CE10	NP_518560.1	Ralstonia solanacearum GMI1000
306	104484	CE10	NP_593817.1	Schizosaccharomyces pombe 972h-
307	148087	CE10	CAB93516.1	Bacillus sp. BP-7
308	116208	CE10	NP_421101.1	Caulobacter crescentus CB15
309	113168	CE10	NP_518560.1	Ralstonia solanacearum GMI1000
310	71664	CE10	NP_593970.1	Schizosaccharomyces pombe 972h-
311	161426	CE10	NP_518560.1	Ralstonia solanacearum GMI1000
312	184845	CE10	NP_631192.1	Streptomyces coelicolor A3(2)
313	114755	CE10	NP_776464.1	Bos taurus
314	191182	CE10	NP_518560.1	Ralstonia solanacearum GMI1000
315	164562	CE10	NP_725941.1	Drosophila melanogaster
316	164562	CE10	NP_725942.1	Drosophila melanogaster
317	164562	CE10	NP_611463.1	Drosophila melanogaster
318	77714	CE10	NP_127272.1	Pyrococcus abyssi GE5

319	151574	CE11	CAL53384.1	Ostreococcus tauri OTTH0595
320	113344	CE11	CAL53384.1	Ostreococcus tauri OTTH0595
321	81498	CE11	CAL53384.1	Ostreococcus tauri OTTH0595
322	172817	CE11	CAL53384.1	Ostreococcus tauri OTTH0595
323	88521	CE11	CAL53384.1	Ostreococcus tauri OTTH0595
324	116894	CE11	CAL53384.1	Ostreococcus tauri OTTH0595
325	136688	CE11	CAL53384.1	Ostreococcus tauri OTTH0595
326	118649	CE11	CAL53384.1	Ostreococcus tauri OTTH0595
327	128375	CE11	CAL53384.1	Ostreococcus tauri OTTH0595
328	75677	CE11	CAL53384.1	Ostreococcus tauri OTTH0595
329	147613	CE11	CAL53384.1	Ostreococcus tauri OTTH0595
330	136386	CE11	CAL53384.1	Ostreococcus tauri OTTH0595
331	154262	CE11	CAL53384.1	Ostreococcus tauri OTTH0595
332	33636	CE11	CAL53384.1	Ostreococcus tauri OTTH0595
333	73792	CE11	CAL53384.1	Ostreococcus tauri OTTH0595
334	142298	CE11	CAL53384.1	Ostreococcus tauri OTTH0595
335	154852	CE11	CAL53384.1	Ostreococcus tauri OTTH0595
336	152045	CE11	CAL53384.1	Ostreococcus tauri OTTH0595
337	182982	CE11	CAL53384.1	Ostreococcus tauri OTTH0595
338	117946	CE11	CAL53384.1	Ostreococcus tauri OTTH0595
339	48668	CE11	CAL53384.1	Ostreococcus tauri OTTH0595
340	186391	CE11	CAL53384.1	Ostreococcus tauri OTTH0595
341	117517	CE11	CAL53384.1	Ostreococcus tauri OTTH0595
342	128316	CE11	CAL53384.1	Ostreococcus tauri OTTH0595
343	157509	CE11	CAL53384.1	Ostreococcus tauri OTTH0595
344	144233	CE11	CAL53384.1	Ostreococcus tauri OTTH0595
345	165072	CE11	CAL53384.1	Ostreococcus tauri OTTH0595
346	151241	CE11	CAL53384.1	Ostreococcus tauri OTTH0595
347	152354	CE11	CAL53384.1	Ostreococcus tauri OTTH0595
348	88745	CE11	CAL53384.1	Ostreococcus tauri OTTH0595
349	172588	CE11	CAL53384.1	Ostreococcus tauri OTTH0595
350	118565	CE11	CAL53384.1	Ostreococcus tauri OTTH0595
351	153487	CE11	CAL53384.1	Ostreococcus tauri OTTH0595
352	151391	CE11	CAL53384.1	Ostreococcus tauri OTTH0595
353	145959	CE14	CAI91276.1	Toxoplasma gondii
354	106673	CE16	CAP94833.1	Penicillium chrysogenum Wisconsin 54-1255
355	156782	CE16	CBY01773.1	Leptosphaeria maculans v23.1.3
356	81833	CE16	CAK37535.1	Aspergillus niger CBS 513.88
357	82209	CE16	EAA66810.1	Aspergillus nidulans FGSC A4
358	157313	CE16	BAE65038.1	Aspergillus oryzae RIB40
359	112879	CE16	CBY01773.1	Leptosphaeria maculans v23.1.3
360	108619	CE2	ADI05786.1	Streptomyces bingchenggensis BCW-1
361	157263	CE2|CBM13	ADI13078.1	Streptomyces bingchenggensis BCW-1
362	151436	CE3	ADK54828.1	uncultured soil bacterium
363	155363	CE3	ADK54828.1	uncultured soil bacterium
364	131995	CE4	AAW50596.1	Volvariella volvacea
365	79711	CE4	ABK58012.1	Batrachochytrium dendrobatidis JEL197

366	156993	CE4	BAE56160.1	Aspergillus oryzae RIB40
367	155780	CE4	BAB03595.1	Phycomyces blakesleeanus
368	184131	CE4	ACU47361.1	Brucella microti CCM 4915
369	184131	CE4	ABQ61499.1	Brucella ovis ATCC 25840
370	184131	CE4	ABX61443.1	Brucella canis ATCC 23365
371	184131	CE4	ABY37469.1	Brucella suis ATCC 23445
372	184131	CE4	NP_697379.1	Brucella suis 1330
373	156314	CE4	CAJ90973.1	Puccinia graminis f. sp. tritici
374	46792	CE4	BAE56160.1	Aspergillus oryzae RIB40
375	155630	CE4	AAX11701.1	Rhizopus stolonifer
376	155411	CE4	BAE56160.1	Aspergillus oryzae RIB40
377	133575	CE4	AAW50596.1	Volvariella volvacea
378	23450	CE4	ABK58012.1	Batrachochytrium dendrobatidis JEL197
379	108697	CE4	CAP65640.1	Podospora anserina S mat+
380	135734	CE4	BAB03595.1	Phycomyces blakesleeanus
381	155947	CE4	AAW62235.1	Rhizopus circinans MUCL30802
382	140466	CE4	BAB03595.1	Phycomyces blakesleeanus
383	156062	CE4	ABK58012.1	Batrachochytrium dendrobatidis JEL197
384	156744	CE4	BAB03595.1	Phycomyces blakesleeanus
385	162369	CE4	ACF22101.1	Aspergillus nidulans FGSC A4
386	162369	CE4	ACE79177.1	Emericella nidulans
387	104376	CE4	BAB03595.1	Phycomyces blakesleeanus
388	155048	CE4	AAE82862.1	Amylomyces rouxii ATCC 24905
389	156315	CE4	XP_360918.1	Magnaporthe grisea 70-15
390	156524	CE4	BAB03595.1	Phycomyces blakesleeanus
391	47303	CE4	BAE56160.1	Aspergillus oryzae RIB40
392	155566	CE4	AAW62235.1	Rhizopus circinans MUCL30802
393	136560	CE4	BAB03595.1	Phycomyces blakesleeanus
394	121218	CE4	XP_360918.1	Magnaporthe grisea 70-15
395	72854	CE4	BAB03595.1	Phycomyces blakesleeanus
396	156362	CE4	ABO38855.1	Mucor racemosus
397	155262	CE4	BAB03595.1	Phycomyces blakesleeanus
398	110151	CE4	BAE56160.1	Aspergillus oryzae RIB40
399	88516	CE4	BAB03595.1	Phycomyces blakesleeanus
400	113815	CE4	AAW50596.1	Volvariella volvacea
401	155735	CE4	XP_360918.1	Magnaporthe grisea 70-15
402	122340	CE4|CBM18	CAP60162.1	Podospora anserina S mat+
403	35027	CE6	ABJ86882.1	Solibacter usitatus Ellin6076
404	84216	CE8	CAN67070.1	Vitis vinifera
405	140107	CE9	ADK30104.1	Brachyspira pilosicoli 95/1000
406	138271	CE9	ADK30104.1	Brachyspira pilosicoli 95/1000
407	104699	CE9	ADG70601.1	Brachyspira murdochii DSM 12563
408	104699	CE9	ADK30104.1	Brachyspira pilosicoli 95/1000
409	143217	CE9	AAI60548.1	inbred)
410	159264	CE9	ADG70601.1	Brachyspira murdochii DSM 12563
411	161275	CE9	AAI60548.1	inbred)
412	161275	CE9	CBG19248.1	Danio rerio

413	104746	CE9	ADG70601.1	Brachyspira murdochii DSM 12563
414	104746	CE9	ACN85082.1	Brachyspira hyodysenteriae WA1 WA1; ATCC 49526
415	25399	GH1	ABF46302.1	Deinococcus geothermalis DSM 11300
416	155249	GH103	ACD61506.1	Xanthomonas oryzae pv. oryzae PXO99A
417	155249	GH103	BAE67016.1	Xanthomonas oryzae pv. oryzae MAFF 311018
418	155020	GH109	BAH39526.1	Gemmatimonas aurantiaca T-27 T-27 (= NBRC 100505)
419	189023	GH109	ADB36574.1	Spirosoma linguale DSM 74
420	83367	GH109	ACQ79069.1	Beutenbergia cavernae DSM 12333
421	75215	GH12	ABA56022.1	Sinorhizobium meliloti
422	138386	GH13	ADO37723.1	Eubacterium limosum KIST612
423	140364	GH13	CAP83076.1	Penicillium chrysogenum Wisconsin 54-1255
424	106868	GH13	ADD42974.1	Stackebrandtia nassauensis DSM 44728
425	151060	GH13	CAG59721.1	Candida glabrata CBS 138
426	163262	GH13	ADD42974.1	Stackebrandtia nassauensis DSM 44728
427	151062	GH15	XP_363338.1	Magnaporthe grisea 70-15
428	156167	GH15|CBM21	CAY05392.1	Mucor circinelloides
429	72222	GH15|CBM21	BAH09876.1	Rhizopus oryzae NBRC 4785
430	72222	GH15|CBM21	BAH09877.1	Amylomyces rouxii CBS 438.76
431	42682	GH16	AAQ20798.1	Rhizopus oryzae
432	42682	GH16	NP_595680.1	Schizosaccharomyces pombe 972h-
433	156806	GH16	AAQ20798.1	Rhizopus oryzae
434	82616	GH16	XP_365073.1	Magnaporthe grisea 70-15
435	182651	GH16	EAA57990.1	Aspergillus nidulans FGSC A4
436	113904	GH16	CBX96484.1	Leptosphaeria maculans v23.1.3
437	78070	GH16	EAA57990.1	Aspergillus nidulans FGSC A4
438	118964	GH16	ADK37844.1	Pandora neoaphidis
439	85659	GH16	CBX96484.1	Leptosphaeria maculans v23.1.3
440	111971	GH16|CBM18	CAP65735.1	Podospora anserina S mat+
441	158770	GH16|CBM18	BAE62875.1	Aspergillus oryzae RIB40
442	121348	GH17	NP_594455.1	Schizosaccharomyces pombe 972h-
443	162624	GH17	ABP48761.1	Tetrapisispora phaffii DBVPG 6706
444	161190	GH17	BAE54672.1	Aspergillus oryzae RIB40
445	142178	GH17	BAE54672.1	Aspergillus oryzae RIB40
446	144545	GH17	BAE54672.1	Aspergillus oryzae RIB40
447	151819	GH17	BAE54672.1	Aspergillus oryzae RIB40
448	163885	GH17	CAG58735.1	Candida glabrata CBS 138
449	157817	GH18	CBX99002.1	Leptosphaeria maculans v23.1.3
450	155387	GH18	CBX99002.1	Leptosphaeria maculans v23.1.3
451	150210	GH18	CBX99002.1	Leptosphaeria maculans v23.1.3
452	74827	GH18	CBX99002.1	Leptosphaeria maculans v23.1.3
453	154933	GH18	CBX99002.1	Leptosphaeria maculans v23.1.3
454	47580	GH18	CBX99002.1	Leptosphaeria maculans v23.1.3
455	154662	GH18	CAH89501.1	Pongo abelii
456	157309	GH18	CBX99002.1	Leptosphaeria maculans v23.1.3
457	153817	GH18	CBX99002.1	Leptosphaeria maculans v23.1.3
458	156381	GH18	CAJ68298.1	Clostridium difficile 630
459	119261	GH18	CBX99002.1	Leptosphaeria maculans v23.1.3

460	44616	GH18	CBX99002.1	Leptosphaeria maculans v23.1.3
461	74060	GH18	CBX99002.1	Leptosphaeria maculans v23.1.3
462	91342	GH18	CBX99002.1	Leptosphaeria maculans v23.1.3
463	134686	GH18	ADK37846.1	Pandora neoaphidis
464	167506	GH18	ABY40380.1	Pantoea dispersa
465	157445	GH18	CBA62479.1	Clostridium difficile CD196
466	157445	GH18	CBE03719.1	Clostridium difficile R20291
467	157445	GH18	CAJ68298.1	Clostridium difficile 630
468	186509	GH18	CAJ68298.1	Clostridium difficile 630
469	16228	GH18	CBX99002.1	Leptosphaeria maculans v23.1.3
470	128698	GH18	CBX99002.1	Leptosphaeria maculans v23.1.3
471	21558	GH18	CBX99002.1	Leptosphaeria maculans v23.1.3
472	136157	GH18	CBX99002.1	Leptosphaeria maculans v23.1.3
473	81750	GH18	EAA59449.1	Aspergillus nidulans FGSC A4
474	149410	GH18	ABY40380.1	Pantoea dispersa
475	153793	GH18	EAA60949.1	Aspergillus nidulans FGSC A4
476	153793	GH18	BAA35140.2	Emericella nidulans FGSC89
477	135450	GH18	CBX99002.1	Leptosphaeria maculans v23.1.3
478	143387	GH18	CBX99002.1	Leptosphaeria maculans v23.1.3
479	140055	GH18	CAC35202.1	Amanita muscaria AM83
480	104074	GH18	CBX99002.1	Leptosphaeria maculans v23.1.3
481	89734	GH18	CBX99002.1	Leptosphaeria maculans v23.1.3
482	129805	GH18	CBX99002.1	Leptosphaeria maculans v23.1.3
483	155545	GH18	CBA62479.1	Clostridium difficile CD196
484	155545	GH18	CBE03719.1	Clostridium difficile R20291
485	155545	GH18	CAJ68298.1	Clostridium difficile 630
486	31509	GH18	CBX99002.1	Leptosphaeria maculans v23.1.3
487	84151	GH18	CBX99002.1	Leptosphaeria maculans v23.1.3
488	72002	GH18	EAA59449.1	Aspergillus nidulans FGSC A4
489	157129	GH18	EAA59449.1	Aspergillus nidulans FGSC A4
490	129150	GH18	CBX99002.1	Leptosphaeria maculans v23.1.3
491	119251	GH18	CAQ51152.1	Coprinellus congregatus A1 = CC16
492	120189	GH18	CBX99002.1	Leptosphaeria maculans v23.1.3
493	143805	GH18	ABN68578.2	Pichia stipitis CBS 6054
494	189121	GH18	EAA60949.1	Aspergillus nidulans FGSC A4
495	189121	GH18	BAA35140.2	Emericella nidulans FGSC89
496	109857	GH18	XP_362287.1	Magnaporthe grisea 70-15
497	156445	GH18	CBX99002.1	Leptosphaeria maculans v23.1.3
498	84290	GH18	EAA59449.1	Aspergillus nidulans FGSC A4
499	40585	GH18	ABB90389.1	Taiwanofungus camphoratus
500	23145	GH18	CBX99002.1	Leptosphaeria maculans v23.1.3
501	72761	GH18	CBX99002.1	Leptosphaeria maculans v23.1.3
502	181246	GH18	CBX99002.1	Leptosphaeria maculans v23.1.3
503	151855	GH18	CBX99002.1	Leptosphaeria maculans v23.1.3
504	42788	GH18	CBX99002.1	Leptosphaeria maculans v23.1.3
505	113816	GH18	CBX99002.1	Leptosphaeria maculans v23.1.3
506	76909	GH18	CBX99002.1	Leptosphaeria maculans v23.1.3

507	187391	GH18	CBX99002.1	Leptosphaeria maculans v23.1.3
508	144029	GH18	CBX99002.1	Leptosphaeria maculans v23.1.3
509	40343	GH18	CBX99002.1	Leptosphaeria maculans v23.1.3
510	86060	GH18	CBX99002.1	Leptosphaeria maculans v23.1.3
511	130021	GH18	CBX99002.1	Leptosphaeria maculans v23.1.3
512	165846	GH18	CAC35202.1	Amanita muscaria AM83
513	72570	GH18	CBX99002.1	Leptosphaeria maculans v23.1.3
514	40393	GH18	CBX99002.1	Leptosphaeria maculans v23.1.3
515	46970	GH18	CBX99002.1	Leptosphaeria maculans v23.1.3
516	76555	GH18	ADK37846.1	Pandora neoaphidis
517	167180	GH18	EAA59449.1	Aspergillus nidulans FGSC A4
518	157173	GH18	CBX99002.1	Leptosphaeria maculans v23.1.3
519	157038	GH18	CBX99002.1	Leptosphaeria maculans v23.1.3
520	150693	GH18	EAA59449.1	Aspergillus nidulans FGSC A4
521	130546	GH18	ABB90389.1	Taiwanofungus camphoratus
522	37784	GH18	CBX99002.1	Leptosphaeria maculans v23.1.3
523	162744	GH18	EAA59449.1	Aspergillus nidulans FGSC A4
524	115839	GH18	CBX99002.1	Leptosphaeria maculans v23.1.3
525	38698	GH18|CBM19	BAA01021.1	Rhizopus microsporus var. oligosporus IFO 8631
526	147080	GH18|CBM19	BAA01021.1	Rhizopus microsporus var. oligosporus IFO 8631
527	121587	GH18|CBM19	CAG62749.1	Candida glabrata CBS 138
528	78368	GH2	CAL54622.1	Ostreococcus tauri OTTH0595
529	32271	GH2	CBX99479.1	Leptosphaeria maculans v23.1.3
530	16755	GH2	CBX99479.1	Leptosphaeria maculans v23.1.3
531	86506	GH2	CBX99479.1	Leptosphaeria maculans v23.1.3
532	147162	GH2	CBX99479.1	Leptosphaeria maculans v23.1.3
533	153054	GH2	CAL54622.1	Ostreococcus tauri OTTH0595
534	104586	GH20	ADU31943.1	Bacillus cellulosilyticus DSM 2522
535	109933	GH20	ABE82127.1	Medicago truncatula
536	128445	GH20	BAF11315.1	Oryza sativa Japonica Group
537	117999	GH20	ABY83274.1	Solanum lycopersicum
538	74439	GH23	ACV36542.1	Candidatus Accumulibacter phosphatis clade IIA str. UW-1
539	167408	GH23	AAZ47176.1	Dechloromonas aromatica RCB
540	158400	GH23	BAD65358.1	Bacillus clausii KSM-K16
541	164675	GH27	ACT01843.1	Paenibacillus sp. JDR-2
542	135330	GH28	CAX58716.1	Erwinia billingiae Eb661
543	156285	GH28	BAE64464.1	Aspergillus oryzae RIB40
544	105563	GH28	BAE64464.1	Aspergillus oryzae RIB40
545	155087	GH28	ACA48699.1	Rhizopus oryzae YM9901
546	154819	GH28	BAE64464.1	Aspergillus oryzae RIB40
547	112333	GH28	BAE64464.1	Aspergillus oryzae RIB40
548	113662	GH28	BAE64464.1	Aspergillus oryzae RIB40
549	181379	GH28	ACA48699.1	Rhizopus oryzae YM9901
550	104492	GH28	BAE64464.1	Aspergillus oryzae RIB40
551	92262	GH28	BAE64464.1	Aspergillus oryzae RIB40
552	91387	GH28	BAE64464.1	Aspergillus oryzae RIB40
553	148944	GH29	ADJ46991.1	Amycolatopsis mediterranei U32

554	129225	GH29	ADJ46991.1	Amycolatopsis mediterranei U32
555	155465	GH3	ACL38786.1	Arthrobacter chlorophenolicus A6
556	153824	GH3	CAP94355.1	Penicillium chrysogenum Wisconsin 54-1255
557	33615	GH3	CAP58431.2	Rhizomucor miehei NRRL 5282
558	142575	GH3	AAZ28832.1	Colwellia psychrerythraea 34H BAA-681
559	38405	GH3	CAP58431.2	Rhizomucor miehei NRRL 5282
560	153684	GH3	CAP58431.2	Rhizomucor miehei NRRL 5282
561	108925	GH31	ABG05503.1	Rubrobacter xylanophilus DSM 9941
562	85502	GH31	BAA11053.1	Mucor javanicus
563	112467	GH31	BAE62256.1	Aspergillus oryzae RIB40
564	43667	GH31	BAA11053.1	Mucor javanicus
565	174253	GH31	BAE61578.1	Aspergillus oryzae RIB40
566	160014	GH32	AAZ14291.1	Leishmania major strain Friedlin
567	107222	GH32	AAZ14291.1	Leishmania major strain Friedlin
568	104294	GH36	ADU21146.1	Ruminococcus albus 7
569	159286	GH36	CBX94864.1	Leptosphaeria maculans v23.1.3
570	159390	GH36	ADU21146.1	Ruminococcus albus 7
571	137559	GH36	ACM48349.1	Rhizopus sp. ACCC 30795 F78
572	157070	GH37	CAP59879.1	Podospora anserina S mat+
573	159931	GH37	AAX19735.1	Glomus mosseae
574	72966	GH37	AAX19735.1	Glomus mosseae
575	84477	GH37	AAX19735.1	Glomus mosseae
576	151777	GH37	AAX19735.1	Glomus mosseae
577	80654	GH38	CAK37873.1	Aspergillus niger CBS 513.88
578	149517	GH38	CAP86007.1	Penicillium chrysogenum Wisconsin 54-1255
579	161603	GH4	ACX49739.1	uncultured marine bacterium 1n22
580	163725	GH4	ACX49739.1	uncultured marine bacterium 1n22
581	155197	GH4	ACX49739.1	uncultured marine bacterium 1n22
582	93498	GH4	ACX49739.1	uncultured marine bacterium 1n22
583	110430	GH4	ACX49739.1	uncultured marine bacterium 1n22
584	153454	GH4	ACX49739.1	uncultured marine bacterium 1n22
585	135024	GH4	ACX49739.1	uncultured marine bacterium 1n22
586	138737	GH4	ACX49739.1	uncultured marine bacterium 1n22
587	166693	GH4	ACX49739.1	uncultured marine bacterium 1n22
588	111826	GH43	CBX91235.1	Leptosphaeria maculans v23.1.3
589	143537	GH43|GH35	ADG11196.1	Caulobacter segnis ATCC 21756
590	BAD95808	GH45	BAC53988	Rhizopus oryzae
591	BAD95809	GH45	BAC53988	Rhizopus oryzae
592	157172	GH45	EJD02345	F. mediter
593	81074	GH46	CBI44192.1	Bacillus amyloliquefaciens DSM7
594	156336	GH47	CAZ31460.1	Schistosoma mansoni
595	138374	GH47	CAZ31460.1	Schistosoma mansoni
596	152367	GH47	CAZ31460.1	Schistosoma mansoni
597	155783	GH47	CAZ31460.1	Schistosoma mansoni
598	156437	GH47	CAZ31460.1	Schistosoma mansoni
599	106177	GH47	BAA08634.1	Aspergillus phoenicis
600	86670	GH47	CAP94087.1	Penicillium chrysogenum Wisconsin 54-1255

601	155041	GH47	CAZ31460.1	Schistosoma mansoni
602	157125	GH47	CAZ31460.1	Schistosoma mansoni
603	184024	GH47	CAZ31460.1	Schistosoma mansoni
604	78806	GH47	CAZ31460.1	Schistosoma mansoni
605	156566	GH47	CAZ31460.1	Schistosoma mansoni
606	141717	GH47	CAZ31460.1	Schistosoma mansoni
607	32737	GH47	CAZ31460.1	Schistosoma mansoni
608	157272	GH47	CAZ31460.1	Schistosoma mansoni
609	156855	GH47	CAZ31460.1	Schistosoma mansoni
610	118863	GH47	CAH66776.1	Oryza sativa Indica Group
611	118863	GH47	BAF15714.1	Oryza sativa Japonica Group
612	156512	GH47	CBX90714.1	Leptosphaeria maculans v23.1.3
613	74780	GH47	CAZ31460.1	Schistosoma mansoni
614	156136	GH47	CAZ31460.1	Schistosoma mansoni
615	154856	GH47	CAZ31460.1	Schistosoma mansoni
616	142223	GH47	CAY06010.1	Ustilago maydis 521
617	142223	GH47	CAY09770.1	Ustilago maydis 521
618	81962	GH47	ABA54911.1	Coccidioides posadasii
619	81962	GH47	ABB36773.2	Coccidioides posadasii SILVEIRA
620	118739	GH47	BAF75842.1	Cricetulus griseus
621	117726	GH47	CBX97511.1	Leptosphaeria maculans v23.1.3
622	154655	GH47	CAZ31460.1	Schistosoma mansoni
623	160249	GH47	CAZ31460.1	Schistosoma mansoni
624	146367	GH47	AAO52054.2	Dictyostelium discoideum AX4
625	147439	GH47	CAZ31460.1	Schistosoma mansoni
626	148376	GH47	CAZ31460.1	Schistosoma mansoni
627	152345	GH47	CAZ31460.1	Schistosoma mansoni
628	155818	GH47	CAZ31460.1	Schistosoma mansoni
629	45061	GH47	CAZ31460.1	Schistosoma mansoni
630	162982	GH47	CAZ31460.1	Schistosoma mansoni
631	154737	GH47	CAZ31460.1	Schistosoma mansoni
632	184378	GH47	CAZ31460.1	Schistosoma mansoni
633	171777	GH47	CAZ31460.1	Schistosoma mansoni
634	181082	GH5	BAE60253.1	Aspergillus oryzae RIB40
635	75195	GH5	CBY01903.1	Leptosphaeria maculans v23.1.3
636	128967	GH5	CAP80421.1	Penicillium chrysogenum Wisconsin 54-1255
637	115999	GH5	CBY01903.1	Leptosphaeria maculans v23.1.3
638	136231	GH5	ABA54914.1	Coccidioides posadasii
639	14085	GH5	NP_596461.1	Schizosaccharomyces pombe 972h-
640	39521	GH5	AAW41138.1	Cryptococcus neoformans var. neoformans JEC21
641	45261	GH63	CBX90523.1	Leptosphaeria maculans v23.1.3
642	185055	GH65	ADN16786.1	Cyanothece sp. PCC 7822
643	129129	GH72|CBM43	ABF93407.1	Paracoccidioides brasiliensis
644	155149	GH76	EAA63396.1	Aspergillus nidulans FGSC A4
645	152844	GH76	EAA63396.1	Aspergillus nidulans FGSC A4
646	160397	GH76	EAA63396.1	Aspergillus nidulans FGSC A4
647	139628	GH76	EAA63396.1	Aspergillus nidulans FGSC A4

648	106827	GH76	EAA63396.1	Aspergillus nidulans FGSC A4
649	140177	GH76	EAA63396.1	Aspergillus nidulans FGSC A4
650	120424	GH76	EAA63396.1	Aspergillus nidulans FGSC A4
651	34200	GH76	EAA63396.1	Aspergillus nidulans FGSC A4
652	157439	GH76	EAA63396.1	Aspergillus nidulans FGSC A4
653	90838	GH76	EAA63396.1	Aspergillus nidulans FGSC A4
654	155404	GH8	CBL17008.1	Ruminococcus sp. 18P13
655	108819	GH8	CBL17008.1	Ruminococcus sp. 18P13
656	86382	GH81	CAG83291.1	Yarrowia lipolytica CLIB122 CLIB99
657	86382	GH81	XP_501038.1	Yarrowia lipolytica W29
658	139818	GH84	CAZ28708.1	Schistosoma mansoni
659	153721	GH84	CAZ28708.1	Schistosoma mansoni
660	153984	GH84	CAZ28708.1	Schistosoma mansoni
661	108712	GH85	AAW12851.1	Mucor hiemalis
662	106603	GH85	CBH14097.1	(MHOM/CI/86/DAL972)
663	156165	GH9	AAM22492.1	Phanerochaete chrysosporium BKM-F-1767
664	153977	GH9	AAM22492.1	Phanerochaete chrysosporium BKM-F-1767
665	114542	GH9	AAM22492.1	Phanerochaete chrysosporium BKM-F-1767
666	106574	GH9	ACN58963.1	uncultured bacterium BLR10
667	104610	GH9	BAC04648.1	Homo sapiens
668	148022	GH92	EAA62552.1	Aspergillus nidulans FGSC A4
669	105183	GH92	EAA62552.1	Aspergillus nidulans FGSC A4
670	134408	GH92	EAA62552.1	Aspergillus nidulans FGSC A4
671	154250	GH92	EAA62552.1	Aspergillus nidulans FGSC A4
672	151684	GH92	EAA62552.1	Aspergillus nidulans FGSC A4
673	157594	GH99	CAZ29389.1	Schistosoma mansoni
674	106562	GT1	ACJ63222.1	Papio anubis
675	155108	GT1	ACJ72161.1	Pueraria montana var. lobata
676	157276	GT1	ACJ63223.1	Papio anubis
677	187167	GT1	EAA58455.1	Aspergillus nidulans FGSC A4
678	155879	GT1	ACX85640.4	Drosophila melanogaster
679	138054	GT1	AAN77910.1	Ustilago maydis
680	154244	GT1	ACJ72161.1	Pueraria montana var. lobata
681	155037	GT1	BAH60889.1	Colletotrichum lagenarium
682	154479	GT1	ACJ72161.1	Pueraria montana var. lobata
683	142172	GT1	ABL75959.1	Maruca vitrata MNPV
684	90185	GT1	AAN10154.1	Canis lupus familiaris
685	154815	GT1	BAJ08154.1	Bombyx mori p50T
686	151787	GT1	ACJ72161.1	Pueraria montana var. lobata
687	156043	GT1	ACX85640.4	Drosophila melanogaster
688	49883	GT1	ADO28896.1	Ictalurus punctatus
689	49883	GT1	ADO28273.1	Ictalurus furcatus
690	49883	GT1	BAC26271.1	Mus musculus C57BL/6J
691	178213	GT1	ACJ72161.1	Pueraria montana var. lobata
692	168079	GT1	ACJ72161.1	Pueraria montana var. lobata
693	135327	GT1	ABO31321.1	Penicillium chrysogenum
694	135327	GT1	CAP81246.1	Penicillium chrysogenum Wisconsin 54-1255

695	181344	GT1	ACJ72161.1	Pueraria montana var. lobata
696	129228	GT1	AAG21378.1	Macaca mulatta
697	150169	GT1	ACJ72161.1	Pueraria montana var. lobata
698	156058	GT1	EAA57970.1	Aspergillus nidulans FGSC A4
699	39631	GT1	NP_593363.1	Schizosaccharomyces pombe 972h-
700	154801	GT1	EAA58455.1	Aspergillus nidulans FGSC A4
701	167461	GT1	AAD28546.1	Dictyostelium discoideum
702	19365	GT1	AAD28546.1	Dictyostelium discoideum
703	76685	GT1	AAG21377.1	Macaca mulatta
704	107535	GT1	CAH89549.1	Pongo abelii
705	155487	GT1	ACJ72161.1	Pueraria montana var. lobata
706	158639	GT10	ACI33278.1	Salmo salar
707	76473	GT10	CAK50261.1	Apis mellifera carnica
708	188598	GT13	AAI66303.1	Xenopus (Silurana) tropicalis
709	106011	GT14	CAZ28837.1	Schistosoma mansoni
710	151673	GT15	CAR23549.1	Lachancea thermotolerans CBS 6340
711	121098	GT15	EAA63186.1	Aspergillus nidulans FGSC A4
712	153852	GT15	XP_363108.1	Magnaporthe grisea 70-15
713	153852	GT15	CAP65329.1	Podospora anserina S mat+
714	153852	GT15	AAX07685.1	Magnaporthe grisea Y34
715	22237	GT15	CAH01651.1	Kluyveromyces lactis NRRL Y-1140
716	141532	GT15	CAX42698.1	Candida dubliniensis CD36
717	150168	GT15	CAH01651.1	Kluyveromyces lactis NRRL Y-1140
718	156808	GT15	XP_363108.1	Magnaporthe grisea 70-15
719	156808	GT15	AAX07685.1	Magnaporthe grisea Y34
720	157277	GT15	CAX42698.1	Candida dubliniensis CD36
721	157277	GT15	CAY70214.1	Pichia pastoris GS115
722	152471	GT15	CAX42700.1	Candida dubliniensis CD36
723	152471	GT15	CBX93825.1	Leptosphaeria maculans v23.1.3
724	14092	GT15	XP_363108.1	Magnaporthe grisea 70-15
725	14092	GT15	AAX07685.1	Magnaporthe grisea Y34
726	86104	GT15	CAH01651.1	Kluyveromyces lactis NRRL Y-1140
727	147631	GT15	CAH01651.1	Kluyveromyces lactis NRRL Y-1140
728	153686	GT15	NP_014742.1	Saccharomyces cerevisiae S288C
729	166497	GT15	CAP95932.1	Penicillium chrysogenum Wisconsin 54-1255
730	166497	GT15	CAX42700.1	Candida dubliniensis CD36
731	130929	GT15	CAP95932.1	Penicillium chrysogenum Wisconsin 54-1255
732	138891	GT17	CBD28604.1	Zea mays
733	136930	GT2	NP_810096.1	Bacteroides thetaiotaomicron VPI-5482
734	113132	GT2	ADN17640.1	Cyanothece sp. PCC 7822
735	154997	GT2	ABB70406.1	Puccinia graminis f. sp. tritici
736	165975	GT2	ACY97654.1	Thermomonospora curvata DSM 43183
737	154518	GT2	BAF73720.1	Pinctada fucata
738	180798	GT2	AAQ98885.1	Dictyostelium discoideum
739	154030	GT2	ADC49105.1	Bacillus pseudofirmus OF4
740	154996	GT2	AAB84284.1	Ustilago maydis 518
741	146431	GT2	BAE55623.1	Aspergillus oryzae RIB40

742	153656	GT2	ACY97654.1	Thermomonospora curvata DSM 43183
743	40786	GT2	BAF37218.1	Lentinula edodes SB1226
744	40786	GT2	BAF41220.1	Lentinula edodes SB1226
745	45643	GT2	ACY97654.1	Thermomonospora curvata DSM 43183
746	133469	GT2	NP_810096.1	Bacteroides thetaiotaomicron VPI-5482
747	154471	GT2	ABB70410.1	Puccinia graminis f. sp. tritici
748	136314	GT2	BAF73720.1	Pinctada fucata
749	170604	GT2	AAQ92917.1	Filobasidiella neoformans
750	116620	GT2	ABQ08059.1	Mytilus galloprovincialis
751	179665	GT2	BAF73720.1	Pinctada fucata
752	143468	GT2	NP_810096.1	Bacteroides thetaiotaomicron VPI-5482
753	74789	GT2	ACY97654.1	Thermomonospora curvata DSM 43183
754	153241	GT2	ABB70410.1	Puccinia graminis f. sp. tritici
755	153118	GT2	ABB70410.1	Puccinia graminis f. sp. tritici
756	143260	GT2	NP_810096.1	Bacteroides thetaiotaomicron VPI-5482
757	72427	GT2	BAC78196.1	Coprinopsis cinerea OKAYAMA-7
758	154372	GT2	ADN17640.1	Cyanothece sp. PCC 7822
759	168848	GT2	ABB70406.1	Puccinia graminis f. sp. tritici
760	41661	GT2	AAB84285.1	Ustilago maydis 518
761	182379	GT2	CAL47710.1	Equus caballus
762	182379	GT2	CAL47709.1	Oryctolagus cuniculus
763	182379	GT2	NP_037285.1	Rattus norvegicus
764	182379	GT2	CAL47708.1	Oryctolagus cuniculus
765	169747	GT2	BAI61021.1	Methanocella paludicola SANAE
766	141270	GT2	ADN17640.1	Cyanothece sp. PCC 7822
767	76041	GT2	NP_810096.1	Bacteroides thetaiotaomicron VPI-5482
768	113373	GT2	NP_810096.1	Bacteroides thetaiotaomicron VPI-5482
769	85917	GT2	BAA01023.1	Rhizopus microsporus var. oligosporus IFO 8631
770	114551	GT2	CAA67797.1	Mucor circinelloides
771	104542	GT2	ACY08042.1	Gibberella moniliformis
772	145794	GT2	ABB70410.1	Puccinia graminis f. sp. tritici
773	149958	GT2	ABQ08059.1	Mytilus galloprovincialis
774	34756	GT2	ADN17640.1	Cyanothece sp. PCC 7822
775	71463	GT2	BAA01024.1	Rhizopus microsporus var. oligosporus IFO 8631
776	150969	GT2	ABJ88399.1	Solibacter usitatus Ellin6076
777	145641	GT2	ACU57894.1	Chitinophaga pinensis DSM 2588
778	138262	GT2	BAF73720.1	Pinctada fucata
779	77196	GT2	ACY97654.1	Thermomonospora curvata DSM 43183
780	188342	GT2	BAA01024.1	Rhizopus microsporus var. oligosporus IFO 8631
781	35205	GT2	ACY97654.1	Thermomonospora curvata DSM 43183
782	143643	GT2	AAB84285.1	Ustilago maydis 518
783	154965	GT2	CAI65847.1	Emiliania huxleyi virus sp. 86
784	51513	GT2	BAF73720.1	Pinctada fucata
785	86289	GT2	NP_810096.1	Bacteroides thetaiotaomicron VPI-5482
786	114562	GT2	ABB70406.1	Puccinia graminis f. sp. tritici
787	81503	GT2	NP_810096.1	Bacteroides thetaiotaomicron VPI-5482
788	156925	GT2	CAI65847.1	Emiliania huxleyi virus sp. 86

789	40808	GT2	BAA01024.1	Rhizopus microsporus var. oligosporus IFO 8631
790	108920	GT2	ADC49105.1	Bacillus pseudofirmus OF4
791	12920	GT2	EAA64262.1	Aspergillus nidulans FGSC A4
792	154743	GT2	ABC20869.1	Rhodospirillum rubrum ATCC 11170
793	106469	GT2	ACY97654.1	Thermomonospora curvata DSM 43183
794	147962	GT2	ADP19604.1	Achromobacter xylosoxidans A8
795	157403	GT2	ADN17640.1	Cyanothece sp. PCC 7822
796	151786	GT2	CAA67797.1	Mucor circinelloides
797	12948	GT2	ACY08041.1	Gibberella moniliformis
798	12948	GT2	AAD19614.2	Paracoccidioides brasiliensis IVICPB73
799	12948	GT2	AAB71697.1	Cryptococcus neoformans var. grubii H99
800	143338	GT2	AAB84285.1	Ustilago maydis 518
801	139938	GT2	AAB84285.1	Ustilago maydis 518
802	141101	GT2	BAF37218.1	Lentinula edodes SB1226
803	141101	GT2	BAF41220.1	Lentinula edodes SB1226
804	43336	GT2	ADC49105.1	Bacillus pseudofirmus OF4
805	42177	GT2	ABS01822.1	Kineococcus radiotolerans SRS30216
806	119058	GT2	BAF37218.1	Lentinula edodes SB1226
807	119058	GT2	BAF41220.1	Lentinula edodes SB1226
808	116409	GT2	ABB70410.1	Puccinia graminis f. sp. tritici
809	140500	GT2	ABQ08059.1	Mytilus galloprovincialis
810	152094	GT2	ADC49105.1	Bacillus pseudofirmus OF4
811	154715	GT2	ABF18315.1	Aedes aegypti
812	84566	GT2	ABZ10271.1	uncultured marine microorganism HF4000_APKG10K24
813	138564	GT2	BAF42027.1	Pleurotus ostreatus AM-1
814	138564	GT2	BAF41225.1	Pleurotus ostreatus AM-1
815	176325	GT2	NP_810096.1	Bacteroides thetaiotaomicron VPI-5482
816	147829	GT2	BAF42027.1	Pleurotus ostreatus AM-1
817	147829	GT2	BAF41225.1	Pleurotus ostreatus AM-1
818	74496	GT2|GT4	ADQ27848.1	Burkholderia pseudomallei MSHR1950
819	74496	GT2|GT4	ADQ27813.1	Burkholderia pseudomallei MSHR1950
820	74496	GT2|GT4	ADQ27825.1	Burkholderia pseudomallei MSHR139
821	20892	GT2|GT41	ACV01704.1	Cyanothece sp. PCC 8802
822	115756	GT2|GT41|GT4	ABG52805.1	Trichodesmium erythraeum IMS101
823	139750	GT20	BAE63282.1	Aspergillus oryzae RIB40
824	40415	GT20	BAE60220.1	Aspergillus oryzae RIB40
825	141221	GT20	BAE63282.1	Aspergillus oryzae RIB40
826	139789	GT20	BAE63282.1	Aspergillus oryzae RIB40
827	153371	GT20	ACY82593.1	Beauveria bassiana
828	153371	GT20	CBX95941.1	Leptosphaeria maculans v23.1.3
829	156964	GT20	ACY82595.1	Beauveria bassiana
830	115066	GT20	CAK40897.1	Aspergillus niger CBS 513.88
831	154019	GT20	XP_502823.1	Yarrowia lipolytica W29
832	154019	GT20	CBG04469.1	Yarrowia lipolytica CLIB122 CLIB99
833	112766	GT20	BAE63282.1	Aspergillus oryzae RIB40
834	107727	GT21	AAK13500.1	Pneumocystis carinii
835	114676	GT22	ACO62525.1	Micromonas sp. RCC299

836	127973	GT22	NP_568305.1	Arabidopsis thaliana
837	109009	GT22	BAE60659.1	Aspergillus oryzae RIB40
838	145759	GT22	NP_649939.1	Drosophila melanogaster
839	145759	GT22	AAI55513.1	Xenopus (Silurana) tropicalis
840	156278	GT22	ACI49017.1	Caenorhabditis brenneri CB5161
841	189922	GT24	BAE65008.1	Aspergillus oryzae RIB40
842	40082	GT27|CBM13	CAZ34341.1	Schistosoma mansoni
843	143455	GT27|CBM13	CAZ34341.1	Schistosoma mansoni
844	149518	GT27|CBM13	CAZ34341.1	Schistosoma mansoni
845	135380	GT3	AAW45795.1	Cryptococcus neoformans var. neoformans JEC21
846	141196	GT3	AAW45795.1	Cryptococcus neoformans var. neoformans JEC21
847	107657	GT30	CBA03971.1	Neisseria meningitidis alpha275
848	90680	GT30	CBA03971.1	Neisseria meningitidis alpha275
849	126447	GT31	CAN60055.1	Vitis vinifera
850	154371	GT31	CAN60055.1	Vitis vinifera
851	126456	GT31	CAN60055.1	Vitis vinifera
852	107752	GT31	CAN60055.1	Vitis vinifera
853	34404	GT31	CAN60055.1	Vitis vinifera
854	110472	GT31	CAJ84709.1	Ciona intestinalis
855	105726	GT31	CAJ84708.1	Canis lupus familiaris
856	157502	GT31	CAN60055.1	Vitis vinifera
857	110650	GT31	CAN60055.1	Vitis vinifera
858	186563	GT31	CAN60055.1	Vitis vinifera
859	183942	GT32	NP_001154561.1	Arabidopsis thaliana
860	114029	GT32	ACB93746.1	Beijerinckia indica subsp. indica ATCC 9039
861	129928	GT33	EAA62506.1	Aspergillus nidulans FGSC A4
862	179406	GT34	EAA66095.1	Aspergillus nidulans FGSC A4
863	110991	GT34	EAA66095.1	Aspergillus nidulans FGSC A4
864	115216	GT34	EAA66095.1	Aspergillus nidulans FGSC A4
865	139614	GT34	EAA66095.1	Aspergillus nidulans FGSC A4
866	157548	GT34	EAA66095.1	Aspergillus nidulans FGSC A4
867	185607	GT34	EAA66095.1	Aspergillus nidulans FGSC A4
868	108970	GT34	EAA66095.1	Aspergillus nidulans FGSC A4
869	146080	GT34	EAA66095.1	Aspergillus nidulans FGSC A4
870	44516	GT34	EAA66095.1	Aspergillus nidulans FGSC A4
871	76222	GT34	CBY00245.1	Leptosphaeria maculans v23.1.3
872	12081	GT34	EAA66095.1	Aspergillus nidulans FGSC A4
873	37717	GT34	EAA66095.1	Aspergillus nidulans FGSC A4
874	89913	GT34	ACE60602.1	Coffea arabica
875	144434	GT34	EAA66095.1	Aspergillus nidulans FGSC A4
876	112602	GT34	EAA66095.1	Aspergillus nidulans FGSC A4
877	161811	GT34	EAA66095.1	Aspergillus nidulans FGSC A4
878	42629	GT34	EAA66095.1	Aspergillus nidulans FGSC A4
879	157402	GT34	EAA66095.1	Aspergillus nidulans FGSC A4
880	107637	GT34	EAA66095.1	Aspergillus nidulans FGSC A4
881	159907	GT34	EAA66095.1	Aspergillus nidulans FGSC A4
882	157161	GT34	CAI11453.1	Nicotiana benthamiana

883	152756	GT35	XP_504982.1	Yarrowia lipolytica W29
884	152756	GT35	CAG77789.1	Yarrowia lipolytica CLIB122 CLIB99
885	173013	GT35	XP_504982.1	Yarrowia lipolytica W29
886	173013	GT35	CAG77789.1	Yarrowia lipolytica CLIB122 CLIB99
887	151650	GT39	EAA62286.1	Aspergillus nidulans FGSC A4
888	151650	GT39	AAK71510.1	Emericella nidulans FGSC26
889	178665	GT39	CAX40804.1	Candida dubliniensis CD36
890	178665	GT39	BAE44795.1	Candida albicans CAF3-1
891	156776	GT39	EAA62286.1	Aspergillus nidulans FGSC A4
892	156776	GT39	AAK71510.1	Emericella nidulans FGSC26
893	141234	GT39	AAW43214.1	Cryptococcus neoformans var. neoformans JEC21
894	151941	GT39	EAA62286.1	Aspergillus nidulans FGSC A4
895	151941	GT39	AAK71510.1	Emericella nidulans FGSC26
896	156139	GT39	AAU09790.1	Pichia angusta DL-1
897	127793	GT39	AAW43214.1	Cryptococcus neoformans var. neoformans JEC21
898	112156	GT39	CAP69590.1	Podospora anserina S mat+
899	146788	GT39	AAW43214.1	Cryptococcus neoformans var. neoformans JEC21
900	160449	GT39	CBX95750.1	Leptosphaeria maculans v23.1.3
901	116926	GT39	AAW43214.1	Cryptococcus neoformans var. neoformans JEC21
902	141912	GT4	ACL49294.1	27774
903	145201	GT4	BAE57910.1	Aspergillus oryzae RIB40
904	149776	GT4	ACL49294.1	27774
905	141797	GT4	ACL25044.1	Chloroflexus aggregans DSM 9485
906	186086	GT4	ACL49294.1	27774
907	148013	GT4	AAW43127.1	Cryptococcus neoformans var. neoformans JEC21
908	106312	GT4	ABG49937.1	Trichodesmium erythraeum IMS101
909	157511	GT4	ADG75248.1	Cellulomonas flavigena DSM 20109
910	117141	GT4	ACL49294.1	27774
911	127304	GT4	ACL49294.1	27774
912	122587	GT4	ACL49294.1	27774
913	155640	GT4	ACL49294.1	27774
914	151412	GT4	ACL49294.1	27774
915	31617	GT4	CAH92114.1	Pongo abelii
916	154315	GT4	BAA34296.1	Rhizomucor pusillus
917	166009	GT4	ACL49294.1	27774
918	147605	GT4	ADJ49655.1	Amycolatopsis mediterranei U32
919	156700	GT4	AAW43127.1	Cryptococcus neoformans var. neoformans JEC21
920	142239	GT4	ACL49294.1	27774
921	161951	GT4	ACL25044.1	Chloroflexus aggregans DSM 9485
922	139417	GT4	ACL49294.1	27774
923	155859	GT4	ADG75248.1	Cellulomonas flavigena DSM 20109
924	83305	GT4	ACL49294.1	27774
925	152567	GT4	ACL49294.1	27774
926	178998	GT4	ACL49294.1	27774
927	179185	GT4	ACL49294.1	27774
928	137671	GT4	ACL49294.1	27774
929	147376	GT4	ABO97838.1	Ostreococcus lucimarinus CCE9901

930	147950	GT4	ACL49294.1	27774
931	149394	GT4	ACL49294.1	27774
932	178464	GT4	ACL49294.1	27774
933	138189	GT4	ACL49294.1	27774
934	135137	GT4	ACL49294.1	27774
935	105158	GT4	ABU56167.1	Roseiflexus castenholzii DSM 13941
936	152868	GT4	ACL49294.1	27774
937	151718	GT4	ACL49294.1	27774
938	31978	GT4	ACL49294.1	27774
939	150597	GT4	ACL49294.1	27774
940	120577	GT4	AAW45761.1	Cryptococcus neoformans var. neoformans JEC21
941	145356	GT4	ACL49294.1	27774
942	158611	GT4	ACL49294.1	27774
943	152959	GT4	ACL49294.1	27774
944	135002	GT4	ACL49294.1	27774
945	117651	GT4	ACL49294.1	27774
946	48059	GT4	ACL49294.1	27774
947	71324	GT4	ACL49294.1	27774
948	110439	GT4	ACL49294.1	27774
949	139702	GT4	ACL49294.1	27774
950	159338	GT4	ABO97838.1	Ostreococcus lucimarinus CCE9901
951	151396	GT4	EAA65179.1	Aspergillus nidulans FGSC A4
952	151396	GT4	NP_595519.1	Schizosaccharomyces pombe 972h-
953	107951	GT4	ABU56167.1	Roseiflexus castenholzii DSM 13941
954	145852	GT4	ACL49294.1	27774
955	165856	GT41	ABB28003.1	Chlorobium chlorochromatii CaD3
956	135172	GT41	CAO90694.1	Microcystis aeruginosa PCC 7806
957	188136	GT41	ABO99001.1	Ostreococcus lucimarinus CCE9901
958	43928	GT41	AAZ58723.1	Prochlorococcus marinus str. NATL2A
959	116819	GT41	CAK37011.1	Aspergillus niger CBS 513.88
960	151858	GT41	AAI63923.1	Danio rerio
961	143322	GT41	ACO61142.1	Micromonas sp. RCC299
962	145020	GT41	BAI90485.1	Arthrospira platensis NIES-39
963	142330	GT41	CAX25218.1	Methylobacterium dichloromethanicum DM4
964	127991	GT41	AAH90599.1	Xenopus (Silurana) tropicalis
965	127991	GT41	AAH82353.1	Xenopus laevis
966	147886	GT41	AAI63923.1	Danio rerio
967	10987	GT41	ADL55111.1	Gallionella capsiferriformans ES-2
968	127394	GT41	ACO61142.1	Micromonas sp. RCC299
969	168799	GT47	CAN80640.1	Vitis vinifera
970	112489	GT47	BAF26162.1	Oryza sativa Japonica Group
971	135619	GT48	AAD45326.2	Coccidioides posadasii SILVEIRA
972	152798	GT48	ABL63820.1	Exophiala dermatitidis
973	153484	GT48	AAW47202.1	Cryptococcus neoformans var. neoformans JEC21
974	153484	GT48	AAD11794.1	Cryptococcus neoformans var. grubii H99
975	109973	GT49	AAO51855.1	Dictyostelium discoideum AX4
976	83771	GT49	AAO51855.1	Dictyostelium discoideum AX4

977	75944	GT49	ACI65301.1	Phaeodactylum tricornutum CCAP 1055/1
978	79106	GT49|GT8	AAU12249.1	Gallus gallus
979	129816	GT49|GT8	ADI96200.1	Mastomys erythroleucus
980	129816	GT49|GT8	CAI29669.1	Pongo abelii
981	129816	GT49|GT8	ADI96193.1	Mastomys natalensis
982	129816	GT49|GT8	ADI96194.1	Mastomys natalensis
983	110467	GT49|GT8	ADI96189.1	Mastomys natalensis
984	110467	GT49|GT8	ADI96200.1	Mastomys erythroleucus
985	110467	GT49|GT8	ADI96195.1	Mastomys erythroleucus
986	110467	GT49|GT8	ADI96191.1	Mastomys natalensis
987	110467	GT49|GT8	ADI96194.1	Mastomys natalensis
988	110467	GT49|GT8	ADI96193.1	Mastomys natalensis
989	110467	GT49|GT8	ADI96196.1	Mastomys erythroleucus
990	110467	GT49|GT8	ADI96199.1	Mastomys erythroleucus
991	110467	GT49|GT8	ADI96201.1	Mastomys erythroleucus
992	158170	GT49|GT8	ADI96179.1	Mastomys erythroleucus
993	141430	GT49|GT8	ADI96189.1	Mastomys natalensis
994	141430	GT49|GT8	ADI96184.1	Mastomys kollmannspergeri
995	141430	GT49|GT8	ADI96200.1	Mastomys erythroleucus
996	141430	GT49|GT8	ADI96195.1	Mastomys erythroleucus
997	141430	GT49|GT8	ADI96191.1	Mastomys natalensis
998	141430	GT49|GT8	ADI96194.1	Mastomys natalensis
999	141430	GT49|GT8	ADI96187.1	Mastomys kollmannspergeri
1000	141430	GT49|GT8	ADI96196.1	Mastomys erythroleucus
1001	141430	GT49|GT8	ADI96193.1	Mastomys natalensis
1002	141430	GT49|GT8	ADI96199.1	Mastomys erythroleucus
1003	147461	GT49|GT8	AAU12249.1	Gallus gallus
1004	166012	GT49|GT8	ADI96189.1	Mastomys natalensis
1005	166012	GT49|GT8	ADI96184.1	Mastomys kollmannspergeri
1006	166012	GT49|GT8	ADI96195.1	Mastomys erythroleucus
1007	166012	GT49|GT8	ADI96191.1	Mastomys natalensis
1008	166012	GT49|GT8	ADI96194.1	Mastomys natalensis
1009	166012	GT49|GT8	ADI96187.1	Mastomys kollmannspergeri
1010	166012	GT49|GT8	ADI96193.1	Mastomys natalensis
1011	166012	GT49|GT8	ADI96196.1	Mastomys erythroleucus
1012	166012	GT49|GT8	ADI96199.1	Mastomys erythroleucus
1013	166012	GT49|GT8	ADI96201.1	Mastomys erythroleucus
1014	116253	GT5	ABD01633.1	Synechococcus sp. JA-2-3Ba(2-13)
1015	156302	GT5	CAL64851.1	Streptomyces glaucescens GLA.O
1016	113879	GT5	ABO94079.1	Ostreococcus lucimarinus CCE9901
1017	148140	GT50	CAN63857.1	Vitis vinifera
1018	37192	GT50	CAJ83771.1	Xenopus (Silurana) tropicalis
1019	121058	GT55	XP_362336.1	Magnaporthe grisea 70-15
1020	124205	GT55	XP_362336.1	Magnaporthe grisea 70-15
1021	187164	GT57	CAK38969.1	Aspergillus niger CBS 513.88
1022	114182	GT57	XP_502922.1	Yarrowia lipolytica W29
1023	163419	GT57	CBX97514.1	Leptosphaeria maculans v23.1.3

1024	108715	GT57	CBX97514.1	Leptosphaeria maculans v23.1.3
1025	166063	GT57	CAA91145.2	Caenorhabditis elegans Bristol N2
1026	108484	GT58	CAL57845.1	Ostreococcus tauri OTTH0595
1027	141572	GT58	AAI64529.1	Danio rerio
1028	157132	GT58	CAL57845.1	Ostreococcus tauri OTTH0595
1029	138289	GT59	AAI19909.1	Bos taurus
1030	153524	GT62	CAP73945.1	Podospora anserina S mat+
1031	151364	GT62	CAP73945.1	Podospora anserina S mat+
1032	168154	GT62	CAP73945.1	Podospora anserina S mat+
1033	155763	GT62	CAP73945.1	Podospora anserina S mat+
1034	142437	GT62	AAK40024.1	Pichia angusta DL1-L
1035	37491	GT62	CAP73945.1	Podospora anserina S mat+
1036	106481	GT64	ADE77623.1	Picea sitchensis
1037	187400	GT66	BAE65548.1	Aspergillus oryzae RIB40
1038	171852	GT67	CAM41416.1	Leishmania braziliensis
1039	150891	GT67	CAM41416.1	Leishmania braziliensis
1040	161513	GT67	CAM41416.1	Leishmania braziliensis
1041	107109	GT71	AAW41452.1	Cryptococcus neoformans var. neoformans JEC21
1042	129990	GT71	EAA53843.1	Magnaporthe grisea 70-15
1043	105710	GT71	AAW41452.1	Cryptococcus neoformans var. neoformans JEC21
1044	161614	GT71	CAP80794.1	Penicillium chrysogenum Wisconsin 54-1255
1045	156380	GT71	EAA53843.1	Magnaporthe grisea 70-15
1046	155971	GT76	NP_172652.2	Arabidopsis thaliana
1047	155892	GT77	ABC67744.1	Dictyostelium discoideum AX3
1048	104770	GT77	ABC67744.1	Dictyostelium discoideum AX3
1049	117918	GT77	ABC67744.1	Dictyostelium discoideum AX3
1050	152493	GT77	ABC67744.1	Dictyostelium discoideum AX3
1051	137416	GT77	ABC67744.1	Dictyostelium discoideum AX3
1052	148495	GT77	ABC67744.1	Dictyostelium discoideum AX3
1053	141212	GT77	ABC67744.1	Dictyostelium discoideum AX3
1054	185119	GT77	ABC67744.1	Dictyostelium discoideum AX3
1055	154168	GT77	ABC67744.1	Dictyostelium discoideum AX3
1056	77324	GT77	ABC67744.1	Dictyostelium discoideum AX3
1057	140255	GT77	ABC67744.1	Dictyostelium discoideum AX3
1058	106385	GT77	ABC67744.1	Dictyostelium discoideum AX3
1059	147969	GT77	ABC67744.1	Dictyostelium discoideum AX3
1060	146824	GT77	ABC67744.1	Dictyostelium discoideum AX3
1061	150039	GT77	ABC67744.1	Dictyostelium discoideum AX3
1062	161913	GT77	ABC67744.1	Dictyostelium discoideum AX3
1063	156660	GT77	ABC67744.1	Dictyostelium discoideum AX3
1064	154150	GT77	ABC67744.1	Dictyostelium discoideum AX3
1065	139462	GT77	ABC67744.1	Dictyostelium discoideum AX3
1066	166142	GT77	ABC67744.1	Dictyostelium discoideum AX3
1067	138281	GT77	ABC67744.1	Dictyostelium discoideum AX3
1068	112695	GT77	ABC67744.1	Dictyostelium discoideum AX3
1069	121605	GT77	ABC67744.1	Dictyostelium discoideum AX3
1070	151123	GT77	ABC67744.1	Dictyostelium discoideum AX3

1071	134984	GT77	ABC67744.1	Dictyostelium discoideum AX3
1072	39757	GT77	ABC67744.1	Dictyostelium discoideum AX3
1073	135961	GT77	ABC67744.1	Dictyostelium discoideum AX3
1074	79140	GT77	ABC67744.1	Dictyostelium discoideum AX3
1075	127850	GT77	ABC67744.1	Dictyostelium discoideum AX3
1076	155372	GT77	ABC67744.1	Dictyostelium discoideum AX3
1077	151495	GT77	ABC67744.1	Dictyostelium discoideum AX3
1078	148263	GT77	ABC67744.1	Dictyostelium discoideum AX3
1079	147429	GT77	ABC67744.1	Dictyostelium discoideum AX3
1080	127981	GT77	ABC67744.1	Dictyostelium discoideum AX3
1081	154683	GT77	ABC67744.1	Dictyostelium discoideum AX3
1082	181840	GT77	ABC67744.1	Dictyostelium discoideum AX3
1083	147761	GT77	ABC67744.1	Dictyostelium discoideum AX3
1084	180601	GT77	ABC67744.1	Dictyostelium discoideum AX3
1085	177085	GT77	ABC67744.1	Dictyostelium discoideum AX3
1086	94625	GT77	ABC67744.1	Dictyostelium discoideum AX3
1087	186072	GT77	ABC67744.1	Dictyostelium discoideum AX3
1088	109602	GT8	AAA31404.1	Oryctolagus cuniculus
1089	148294	GT8	CAZ28559.1	Schistosoma mansoni
1090	186366	GT8	ABW74133.1	Mucor racemosus PTCC 5305
1091	157421	GT8	CAZ34445.1	Schistosoma mansoni
1092	155655	GT9	NP_962186.1	Mycobacterium avium subsp. paratuberculosis K-10
1093	185867	GT9	NP_962186.1	Mycobacterium avium subsp. paratuberculosis K-10
1094	110739	GT90	CAN70737.1	Vitis vinifera
1095	155578	PL14	AAW44587.1	Cryptococcus neoformans var. neoformans JEC21
1096	104534	PL14	ABT15158.1	Paramecium bursaria Chlorella virus NY2A
1097	155219	PL14	AAW44587.1	Cryptococcus neoformans var. neoformans JEC21
1098	114728	PL8	ACB77597.1	Opitutus terrae PB90-1
1099	151055	PL8	ACB77597.1	Opitutus terrae PB90-1
