# Supplementary material for: Genomic, Proteomic, and Biochemical Analyses of Oleaginous Mucor circinelloides: Evaluating Its Capability in Utilizing Cellulolytic Substrates for Lipid Production
Source: PLoS One. 2013 Sep 4;8(9):e71068. doi: 10.1371/journal.pone.0071068 (PMC3762813; doi:10.1371/journal.pone.0071068)
Supplement: Table S2 — List of peptide sequences and proteins identified in the bands of A0–A9 and B0–B8 from concentrator- and ethanol-prepared protein samples, respectively. (PDF) [file pone.0071068.s002.pdf]

Manuscript title: Genomic, proteomic, and biochemical analyses of oleaginous *Mucor circinelloides*: Evaluating its capability in utilizing cellulolytic substrates for lipid production

Supplemental Table S2. List of Peptide sequences and proteins identified in the bands of A0-A9 and B0-B8 from concentrator- and ethanol-prepared protein samples, respectively.

**Band A0**				
Hits	Protein ID	Protein Mass	No. of Peptide	Peptide sequences
1	156167	64906.84	3	ADTFILIADSILK<br>FASLYSINQNLNGYLGNAIGR<br>IDSFWNSNGQYVSVSQSVTGGVSK
**Band A1**				
Hits	Protein ID	Protein Mass	No. of Peptide	
1	155115	37564.85	11	SKNIVGADQFTK<br>TLILITFDENASTSKR<br>TLILITFDENASTSK<br>TLILITFDENASTSK<br>GTTDSTFYTHFSSLSTVEHNWDLGNLGR<br>AFDHILQVWFENQDYSTIAK<br>AFDHILQVWFENQDYSTIAK<br>RNQVWSLLLGNIPANLK<br>AFDHILQVWFENQDYSTIAK<br>NQVWSLLLGNIPANLK<br>NQVWSLLLGNIPANLK
2	156269	42262.39	3	SWSIQYGDGSTASGILAK<br>TPVDNLISQGLISSPVFGVWLGK<br>AGTTSVASSFSGILDTGTTLLLFTQSVASK
**Band A2**				
Hits	Protein ID	Protein Mass	No. of Peptide	
1	156269	42262.39	12	NNYVVFNQK<br>NNYVVFNQK<br>SWSIQYGDGSTASGILAK<br>DTVNLGGLVIK<br>ASNGGGGEYLFGGSNPNHYTGALTTVPVDK<br>TPVDNLISQGLISSPVFGVWLGK<br>TPVDNLISQGLISSPVFGVWLGK<br>TPVDNLISQGLISSPVFGVWLGK<br>AGTTSVASSFSGILDTGTTLLLFTQSVASK<br>AGTTSVASSFSGILDTGTTLLLFTQSVASK<br>ESSSFASDPIDGLMGLGFDTITTVAGIK<br>ESSSFASDPIDGLMGLGFDTITTVAGIK
2	155115	37564.85	7	VYQESIPSVGYTGYK<br>TLILITFDENASTSK<br>GTTDSTFYTHFSSLSTVEHNWDLGNLGR<br>GTTDSTFYTHFSSLSTVEHNWDLGNLGR<br>AFDHILQVWFENQDYSTIAK<br>RNQVWSLLLGNIPANLK<br>NQVWSLLLGNIPANLK
3	BAD95808	34332.99	2	YYSQCLPGSHSNNAGNASSTK<br>TSTTAAASTSTSSSAGYK
**Band A3**				
Hits	Protein ID	Protein Mass	No. of Peptide	
1	156806	34114.32	2	GLIYTQNNQVIIK<br>SNAPNPNNWGTPMAAFPFVSGK
2	155646	18948.17	1	SAHYASTANYQQVTGR

**Band A4**				
Hits	Protein ID	Protein Mass	No. of Peptide	
1	115037	20656.54	19	TSGYAQVTGTIDR<br>TSGYAQVTGTIDR<br>TSGYAQVTGTIDR<br>TSGYAQVTGTIDR<br>TSGYAQVTGTIDR<br>TSGYAQVTGTIDR<br>TSGYAQVTGTIDR<br>TSGYAQVTGTIDR<br>TSGYAQVTGTIDR<br>TSGYAQVTGTIDR<br>AAYQLDPNDGGGQYDNMDIK<br>TSGYAQVTGTIDR<br>AAYQLDPNDGGGQYDNMDIK<br>TAHFVKTSGYAQVTGTIDR<br>TSGYAQVTGTIDR<br>TSGYAQVTGTIDR<br>TSGYAQVTGTIDR<br>TSGYAQVTGTIDR<br>TSGYAQVTGTIDR
2	156806	34114.32	2	GLIYTQNNQVIIK<br>SNAPNPNNWGTPMAAFPFVSGK
3	155646	18948.17	1	SAHYASTANYQQVTGR
**Band A5**				
Hits	Protein ID	Protein Mass	No. of Peptide	
1	115037	20656.54	23	TSGYAQVTGTIDR<br>TSGYAQVTGTIDR<br>TSGYAQVTGTIDR<br>TSGYAQVTGTIDR<br>TSGYAQVTGTIDR<br>TSGYAQVTGTIDR<br>TSGYAQVTGTIDR<br>TSGYAQVTGTIDR<br>TSGYAQVTGTIDR<br>TAHFVKTSGYAQVTGTIDR<br>TSGYAQVTGTIDR<br>TSGYAQVTGTIDR<br>AAYQLDPNDGGGQYDNMDIK<br>TSGYAQVTGTIDR<br>AAYQLDPNDGGGQYDNMDIK<br>TSGYAQVTGTIDR<br>TSGYAQVTGTIDR<br>AAYQLDPNDGGGQYDNMDIK<br>AAYQLDPNDGGGQYDNMDIK<br>AAYQLDPNDGGGQYDNMDIK<br>AAYQLDPNDGGGQYDNMDIK<br>TSGYAQVTGTIDR<br>TSGYAQVTGTIDR
2	155817	41544.01	2	AVVSDYFPAVQSQLTAYPGYK<br>VGNPAFAYYVDSTGITFSR
3	155646	18948.17	1	SAHYASTANYQQVTGR

Band A6				
Hits		Protein Mass	No. of Peptide	
1	155646	18948.17	6	SAHYASTANYQQVTGR<br>SAHYASTANYQQVTGR<br>SAHYASTANYQQVTGR<br>SAHYASTANYQQVTGR<br>SAHYASTANYQQVTGR<br>SAHYASTANYQQVTGR
2	191377	11741.29	3	HQNGVIAGSVAPGGSAGSR<br>ISPPVTLR<br>APTWLQVTIFEDIDFGGASHVYK
3	86506	23626.97	2	SIQVNQLTNTVGNHK<br>ATTLAAGSSTSINVDSTWGGR
4	92193	19499.31	1	KPQFDAILLATTLVK
5	156103	14879.71	1	WETTYNDGTGQQVPIPDGYK

Band A7				
Hits	Protein ID	Protein Mass	No. of Peptide	
1	191377	11741.29	63	HQNGVIAGSVAPGGSAGSR<br>HQNGVIAGSVAPGGSAGSR<br>HQNGVIAGSVAPGGSAGSR<br>HQNGVIAGSVAPGGSAGSR<br>HQNGVIAGSVAPGGSAGSR<br>APTWLQVTIFEDIDFGGASHVYKGPQKK<br>APTWLQVTIFEDIDFGGASHVYKGPQK<br>APTWLQVTIFEDIDFGGASHVYK

				[illegible]<br>APTWLQVTIFEDIDFGGASHVYK
2	157923	18919.85	4	SAGYTGALAVHSSEQTLETK<br>SAGYTGALAVHSSEQTLETK<br>SAGYTGALAVHSSEQTLETK<br>KPEFDAVLLATSLVK
3	155646	18948.17	4	SAHYASTANYQQVTGR<br>SAHYASTANYQQVTGR<br>SAHYASTANYQQVTGR<br>SAHYASTANYQQVTGR
4	115037	20656.54	3	TSGYAQVTGTIDR<br>TSGYAQVTGTIDR<br>AAYQLDPNDGGGQYDNMDIK
5	92193	19499.31	3	TPATHLDAANNLVNQINTSFATAK<br>KPQFDAILLATTLVK<br>KPQFDAILLATTLVK
6	157172	18447.65	3	AIDLTPAAFQK<br>AIDLTPAAFQK<br>LATLNDGVVDISWK
7	156103	14879.71	3	LGYLENNDPYNEHLK<br>WETTYNDGTGQQVPIPDGYK<br>DFALNAGSQTITLPADLETK
8	157488	30733.9	1	TITGLDLYSVDASGNPK
9	184762	17351.86	1	VSDSHVVNDAQLVLTNTK

Band A8				
Hits	Protein ID	Protein Mass	No. of Peptide	
1	156103	14879.71	28	AGQSYVVK<br>AGQSYVVK<br>FTIQAAR<br>LGYLENNDPYNEHLK<br>LGYLENNDPYNEHLK<br>LGYLENNDPYNEHLK<br>LGYLENNDPYNEHLK<br>LGYLENNDPYNEHLK<br>LGYLENNDPYNEHLK<br>LGYLENNDPYNEHLK<br>WETTYNDGTGQQVPIPDGYK<br>WETTYNDGTGQQVPIPDGYK<br>WETTYNDGTGQQVPIPDGYKGTIK<br>WETTYNDGTGQQVPIPDGYKGTIK<br>WETTYNDGTGQQVPIPDGYK<br>WETTYNDGTGQQVPIPDGYKGTIK<br>WETTYNDGTGQQVPIPDGYK<br>AGQSYVVKWETTYNDGTGQQVPIPDGYKGTIK<br>AGQSYVVKWETTYNDGTGQQVPIPDGYK<br>TSYIIVLMGNSGNASPK<br>TSYIIVLMGNSGNASPK<br>DFALNAGSQTITLPADLETK<br>DFALNAGSQTITLPADLETK<br>DFALNAGSQTITLPADLETK<br>DFALNAGSQTITLPADLETK<br>DFALNAGSQTITLPADLETK<br>FGDLAKDFALNAGSQTITLPADLETK<br>FGDLAKDFALNAGSQTITLPADLETK
2	191377	11741.29	9	HQNGVIAGSVAPGGSAGSR<br>HQNGVIAGSVAPGGSAGSR<br>HQNGVIAGSVAPGGSAGSR<br>APTWLQVTIFEDIDFGGASHVYKGPQK<br>APTWLQVTIFEDIDFGGASHVYK<br>APTWLQVTIFEDIDFGGASHVYK<br>APTWLQVTIFEDIDFGGASHVYK<br>APTWLQVTIFEDIDFGGASHVYK<br>APTWLQVTIFEDIDFGGASHVYK
3	156010	30340.93	3	KITGLDLYSVDSAGK<br>YVQNVWSGSYALNTK<br>ITGLDLYSVDSAGK
4	156218	18949.67	3	SHLSSFDDKTSR<br>SKEGSYNFLTK<br>VLNALNTIK
5	157285	16118.75	3	TPYFHVEYNK<br>SWVSVDLYEYK<br>ASGISTPWFNVDLGK
6	157923	18919.85	2	SAGYTGALAVHSSEQTLETK<br>KPEFDAVLLATSLVK
7	155646	18948.17	1	SAHYASTANYQQVTGR
8	157172	18447.65	1	LATLNDGVVDISWK
**Band A9**				
Hits	Protein ID	Protein Mass	No. of Peptide	
1	156103	14879.71	3	LGYLENNDPYNEHLK<br>WETTYNDGTGQQVPIPDGYK<br>DFALNAGSQTITLPADLETK
2	184762	17351.86	1	VSDSHVVNDAQLVLTNTK

**Band B0**				
Hits	Protein ID	Protein Mass	No. of Peptide	Peptide sequences
1	38405	78552.88	2	FASNVSSGVSGINAAASWDK<br>GWESGGEDPYLQGVTAAETIK
**Band B1**				
Hits	Protein ID	Protein Mass	No. of Peptide	Peptide sequences
1	156167	64906.84	33	ADTFILIADSILK<br>ADTFILIADSILK<br>ADTFILIADSILK<br>ADTFILIADSILK<br>AIKEWFNNGGVTVTSISLNFFK<br>AIKEWFNNGGVTVTSISLNFFKK<br>ATTYTNTAASIK<br>ATTYTNTAASIK<br>DLTWSHASLISASYAK<br>DLTWSHASLISASYAK<br>EWFNNGGVTVTSISLNFFK<br>FASLYSINQNLNGYLGNAIGR<br>FASLYSINQNLNGYLGNAIGR<br>FASLYSINQNLNGYLGNAIGR<br>FASLYSINQNLNGYLGNAIGR<br>FASLYSINQNLNGYLGNAIGR<br>FDSSAAVGTK<br>IDSFWNSNGQYVSVSQSVTGGVSK<br>IDSFWNSNGQYVSVSQSVTGGVSK<br>IDSFWNSNGQYVSVSQSVTGGVSK<br>IDSFWNSNGQYVSVSQSVTGGVSK<br>QTGDATYVTGTLAPAIYK<br>QTGDATYVTGTLAPAIYK<br>SFTYDGSTLAGQIYIK<br>SFTYDGSTLAGQIYIK<br>SFTYDGSTLAGQIYIK<br>SFTYDGSTLAGQIYIK<br>TKIDSFWNSNGQYVSVSQSVTGGVSK<br>TKIDSFWNSNGQYVSVSQSVTGGVSK<br>TKIDSFWNSNGQYVSVSQSVTGGVSK<br>YTVGTSSFNSLVQNVAVAADAFFSTIK<br>YTVGTSSFNSLVQNVAVAADAFFSTIK<br>YTVGTSSFNSLVQNVAVAADAFFSTIK
**Band B2**				
Hits	Protein ID	Protein Mass	No. of Peptide	Peptide Sequence
1	155115	37564.85	7	VYQESIPSVGYTGYK<br>TLILITFDENASTSKR<br>TLILITFDENASTSK<br>GTTDSTFYTHFSSLSTVEHNWDLGNLGR<br>AFDHILQVWFENQDYSTIAK<br>RNQVWSLLLGNIPANLK<br>NQVWSLLLGNIPANLK
2	84529	31936.78	1	VAQNVASAVQDIITK
**Band B3**				
Hits	Protein ID	Protein Mass	No. of Peptide	
1	84529	31936.78	8	DVSIPVENHGYGGATTNNADAYSK<br>VVVFADSYTDNGNDYK<br>FNGWTVPGLK<br>VHAAWGAWALENLKK<br>VAQNVASAVQDIITK<br>VHAAWGAWALENLK<br>EFAFFSVAPFDK<br>EFAFFSVAPFDKWPVIK

2	155115	37564.85	7	VYQESIPSVGYTGYK<br>TLILITFDENASTSKR<br>TLILITFDENASTSK<br>AFDHILQVWFENQDYSTIAK<br>RNQVWSLLLGNIPANLK<br>NQVWSLLLGNIPANLK<br>NQVWSLLLGNIPANLK
3	115037	20656.54	4	TSGYAQVTGTIDR<br>TSGYAQVTGTIDR<br>TSGYAQVTGTIDR<br>AAYQLDPNDGGGQYDNMDIK
4	155048	46677.27	1	TNQEVLAEFYYTQK
5	181379	41360.8	1	SLTISNVVINDITGSVSEK
**Band B4**				
Hits	Protein ID	Protein Mass	No. of Peptide	
1	84529	31936.78	19	DVSIPVENHGYGGATTNNADAYSK<br>DVSIPVENHGYGGATTNNADAYSK<br>NSEFPPSPPYYAGR<br>NSEFPPSPPYYAGR<br>VVVFADSYTDNGNDYK<br>VVVFADSYTDNGNDYK<br>NSEFPPSPPYYAGR<br>KMIEDYNSYVEGEIK<br>MIEDYNSYVEGEIK<br>NSEFPPSPPYYAGR<br>HFFWDSYHPEK<br>NSEFPPSPPYYAGR<br>VHAAWGAWALENLKK<br>VAQNVASAVQDIITK<br>KVHAAWGAWALENLK<br>FSNGPTWLEYVAK<br>VHAAWGAWALENLK<br>EFAFFSVAPFDK<br>EFAFFSVAPFDKWPVIK
2	115037	20656.54	8	TSGYAQVTGTIDR<br>TSGYAQVTGTIDR<br>TSGYAQVTGTIDR<br>TSGYAQVTGTIDR<br>TSGYAQVTGTIDR<br>TSGYAQVTGTIDR<br>TSGYAQVTGTIDR<br>AAYQLDPNDGGGQYDNMDIK
3	115625	28451.95	2	DKFDLFGALNLNGVSPGNR<br>YQDVIEYFQQVLDLR
**Band B5**				
Hits	Protein ID	Protein Mass	No. of Peptide	
1	157263	16950.72	7	NGNFVNLHSGK<br>KESSDNENQLWEYR<br>GAEDENGAEVILYEK<br>ESSDNENQLWEYR<br>SGTVAANQQWELVR<br>SGTVAANQQWELVR<br>WLIDHEGYIHISSNPDLVLDIK
2	115037	20656.54	2	TSGYAQVTGTIDR<br>AAYQLDPNDGGGQYDNMDIK
3	191377	11741.29	2	HQNGVIAGSVAPGGSAGSR<br>APTWLQVTIFEDIDFGGASHVYK
4	157488	30733.9	1	TITGLDLYSVDASGNPK

**Band B6**				
Hits	Protein ID	Protein Mass	No. of Peptide	
1	115037	20656.54	3	TSGYAQVTGTIDR<br>TSGYAQVTGTIDR<br>AAYQLDPNDGGGQYDNMDIK
2	156103	14879.71	3	LGYLENNDPYNEHLK<br>WETTYNDGTGQQVPIPDGYK<br>DFALNAGSQTITLPADLETK
3	191377	11741.29	3	HQNGVIAGSVAPGGSAGSR<br>APTWLQVTIFEDIDFGGASHVYK<br>APTWLQVTIFEDIDFGGASHVYK
4	155646	18948.17	1	SAHYASTANYQQVTGR

**Band B7**				
Hits	Protein ID	Protein Mass	No. of Peptide	
1	156103	14879.71	8	LGYLENNDPYNEHLK<br>WETTYNDGTGQQVPIPDGYK<br>WETTYNDGTGQQVPIPDGYK<br>WETTYNDGTGQQVPIPDGYKGTIK<br>TSYIIVLMGNSGNASPK<br>DFALNAGSQTITLPADLETK<br>DFALNAGSQTITLPADLETK<br>FGDLAKDFALNAGSQTITLPADLETK
2	156010	30340.93	2	YVQNVWSGSYALNTK<br>ITGLDLYSVDSAGK
3	155263	2114.11	1	ISPDIVQAGSELTIEASGTVK

**Band B8**				
Hits	Protein ID	Protein Mass	No. of Peptide	
1	156103	14879.71	3	LGYLENNDPYNEHLK<br>WETTYNDGTGQQVPIPDGYK<br>WETTYNDGTGQQVPIPDGYK
